# PHGDH-dependent serine metabolism in astrocytes: A key regulator of oxidative stress and pyroptosis in cerebral ischemia-reperfusion injury

**DOI:** 10.1016/j.redox.2025.103954

**Published:** 2025-11-29

**Authors:** Jimeng Zhang, Yaowen Luo, Changcai Xie, Min Zhang, Heyao Qin, Yuefei Zhou, Xiuquan Wu, Chenchen Hu, Feiming Hu, Xiaowei Fei, Hongchen Zhang, Juan Li, Yihao Fu, Yunchao Yuan, Shuya Yang, Dakuan Gao

**Affiliations:** a Department of Neurosurgery, Xijing Hospital, Air Force Military Medical University, Xi'an, Shaanxi, 710032, China; b Department of Cell Biology, National Translational Science Center for Molecular Medicine, Air Force Military Medical University, Xi'an, Shaanxi, 710032, China; c Department of Immunology, Air Force Military Medical University, Xi'an, Shaanxi, 710032, China

**Keywords:** Cerebral ischemia/reperfusion injury, Astrocyte, Pyroptosis, PHGDH, AIM2 inflammasome, Oxidative stress

## Abstract

Phosphoglycerate dehydrogenase (PHGDH) is a key molecule in the progression of Alzheimer's disease. Herein, we report that PHGDH exerts a significant influence on cerebral ischemia-reperfusion injury (CIRI). Ischemia-reperfusion injury is predominantly triggered by oxidative stress and inflammatory responses; however, the underlying molecular mechanisms remain incompletely understood. Our findings demonstrate that PHGDH displays predominant expression in brain astrocytes and undergoes time-dependent alterations in reactive astrocytes following cerebral ischemia-reperfusion. Specifically, targeted knockdown of PHGDH expression in astrocytes substantially exacerbates pathological damage and neurological deficits post cerebral ischemia-reperfusion. Subsequent mechanistic analyses unveiled that PHGDH knockdown predominantly facilitates astrocyte pyroptosis and neuroinflammation. Specifically, downregulation of PHGDH in astrocytes induces oxidative stress, augments ROS production, and diminishes antioxidant levels of GSH and NADPH. Moreover, PHGDH downregulation disrupts the mitochondrial respiratory chain, triggering mitochondrial damage and dsDNA release during ischemia-reperfusion, thereby exacerbating oxidative stress. Collectively, these mechanisms culminate in AIM2 inflammasome activation, as evidenced by substantial increases in AIM2, ASC, and Cleaved Caspase-1 expression. Notably, exogenous depletion of serine and glycine fails to fully explain the astrocyte pyroptosis triggered by PHGDH knockdown. In conclusion, downregulation of PHGDH in astrocytes post cerebral ischemia-reperfusion predominantly drives astrocyte pyroptosis via oxidative stress, resulting in the release of pro-inflammatory cytokines (e.g., IL-1β and IL-18) and subsequent exacerbation of ischemia-reperfusion injury. These novel insights into the role of PHGDH may inform the development of targeted therapeutic strategies for cerebral ischemia-reperfusion.

## 1 Introduction

Acute ischemic stroke affects millions of individuals worldwide annually, severely compromising human health and life expectancy. Cerebral ischemia-reperfusion injury, a critical pathological mechanism underlying post-ischemic neurological deterioration, encompasses a complex interplay of oxidative stress [1,2], calcium overload [3], inflammatory cascades [4], and programmed cell death. The injury to neurons following stroke and the release of damage-associated molecular patterns (DAMPs) drive the progression of global brain inflammation [5], characterized by the activation of neuroglia, recruitment of peripheral immune cells, and the release of cytokines and chemokines. Astrocytes, as pivotal immune-regulatory cells within the brain, have traditionally been recognized for their functional interactions with resident central nervous system cells, such as microglia [6], or infiltrating cells [7]. Recent studies, however, have highlighted that dysfunctions in astrocytes can significantly influence the pathological course of I/R through the release of inflammatory mediators [8] and impaired mitochondrial transfer [9]. Ischemia-reperfusion injury involves various modes of cell death, among which pyroptosis is one of the most prevalent [10]. Pyroptosis, a recently recognized modality of inflammatory programmed cell death [11], is defined by the release of pro-inflammatory mediators that propagate inflammatory cascades and instigate immune activation. Growing evidence demonstrates that ischemia-reperfusion potently induces pyroptosis in astrocytes. Suppression of pyroptosis has been demonstrated to confer anti-inflammatory and neuroprotective effects. Nevertheless, the precise upstream regulatory mechanisms remain elusive.

Pyroptosis is a caspase-dependent programmed cell death pathway, orchestrated via four distinct mechanisms: the canonical inflammasome pathway, the non-canonical inflammasome pathway, the apoptotic caspases-mediated pathway, and the granzymes-mediated pathway [10]. The canonical inflammasome pathway promotes the expression of AIM2, NLRP3, and other core components, thereby facilitating caspase-1 activation. This activation triggers the proteolytic cleavage of gasdermin D (GSDMD) and maturation of pro-inflammatory cytokines IL-1β and IL-18. The N-terminal fragment of GSDMD undergoes oligomerization to assemble plasma membrane pores, leading to cellular swelling and osmotic lysis. This process enables the release of pro-inflammatory cytokines, including IL-1β and IL-18, thereby exacerbating neuronal injury during the acute phase of ischemia-reperfusion injury [12].

Serine, a non-essential amino acid, exerts critical functions in redox homeostasis maintenance, cell proliferation, and one-carbon metabolism [13]. As a key metabolite in biosynthetic pathways, serine serves as a precursor for the biosynthesis of proteins, nucleic acids, and lipids. As a nutritional supplement, serine contributes to the regulation of metabolic homeostasis and cellular health under pathological or stressful conditions. During oxidative stress or exogenous infection, endogenous serine levels may become inadequate to satisfy cellular requirements, thereby proposing that exogenous supplementation represents a potential therapeutic strategy [14,15]. As a rate-limiting enzyme in de novo serine biosynthesis, PHGDH catalyzes the oxidation of the glycolytic intermediate 3-phosphoglycerate to 3-phosphohydroxypyruvate, which is then transaminated to serine. The serine de novo synthesis pathway (SSP) is of paramount importance for energy metabolism, as it constitutes approximately 50 % of the anaplerotic flux into the tricarboxylic acid (TCA) cycle. Even in the presence of exogenous serine, pharmacological inhibition of PHGDH potently suppresses cell proliferation and disrupts metabolic homeostasis [16]. Dysregulation of the SSP, resulting in inadequate endogenous serine biosynthesis, has been linked to multiple pathological conditions, such as Alzheimer's disease [17], psoriasis [18], and acute pancreatitis [19]. The PHGDH-regulated SSP is also intricately linked to the immune system. Serine metabolism modulates macrophage polarization [20,21], regulates inflammatory cytokine secretion [22], and influences antiviral innate immune responses [23]. Under glucose-deprived conditions, PHGDH induces p53 activation, thereby promoting apoptotic cell death [24]. These findings highlight the pivotal role of the PHGDH-mediated SSP in neurodegenerative diseases, inflammatory disorders, and cell fate decisions under pathological and stressful conditions.

In the present study, we documented a significant suppression of PHGDH-dependent serine metabolic pathways after cerebral ischemia-reperfusion, coupled with a substantial decrease in PHGDH expression. Using genetic manipulation of PHGDH expression, integrated transcriptomic and metabolomic analyses, and experimental validation, we herein report that PHGDH downregulation after I/R induces pyroptosis via oxidative stress in astrocytes. Therefore, the current investigation aims to elucidate the molecular mechanisms by which PHGDH-mediated downregulation of serine metabolism initiates astrocyte - associated oxidative stress, pyroptosis, and subsequently exacerbates neuronal injury following cerebral ischemia-reperfusion.

## 2 Methods

### 2.1 Animals

All animal experiments were conducted in accordance with protocols approved by the Institutional Ethics Committee of Xijing Hospital. Adult male C57BL/6 mice (8–12 weeks old) were purchased from the Animal Experimental Center of Air Force Medical University. Upon arrival, the animals underwent a one-week acclimatization period, during which they were housed in a pathogen-free controlled environment. The mice were kept in polycarbonate cages with standard wood chip bedding. They were maintained under controlled conditions, with a temperature of 22 ± 2 °C, humidity of 55 ± 10 %, and a 12-h light/dark cycle (lights on from 08:00 to 20:00). Subjects were provided with laboratory chow and had unrestricted access to water as needed. The food was irradiated, the water was filtered, and the cage equipment was sterilized. All scientific investigations involving animals were conducted in accordance with the ARRIVE (Animal Research: Reporting of In Vivo Experiments) guidelines. All experimental protocols were approved by the Institutional Animal Care and Use Committee of Air Force Medical University. The number of animals assigned to each group was determined based on numbers reported in published studies or from our previous experiments, and the exact number of animals is included in the figure legends. Animals were randomly assigned to different groups using a random number generator. The experiments were conducted by researchers who were blinded to the group assignments.

### 2.2 Lentivirus and adeno-associated virus

Astrocytes were subjected to PHGDH knockdown (shPHGDH) and PHGDH overexpression (LV-PHGDH^OE^) via lentivirus (hU6-MCS-CBh-gcGFP-IRES-Puromycin and hEF1/HTLVp-EGFP-P2A-Puromycin) infection.

Mice were infected with adeno-associated virus (AAV) to knock down PHGDH (AAV9-GFAP-siPHGDH) or overexpression (AAV9-GFAP-PHGDH) in the hippocampal region of the ischemic penumbra.

GenBank ID of the overexpression plasmids were NM_016966.3(T57/78A) and NM_016966.3(R135W/V261 M). The construction of the aforementioned lentivirus, AAV, and plasmids was completed with the assistance of GeneChem Co., Inc. (Shanghai, China).

A schematic diagram illustrating the specific vector element construction is provided in the supplementary figure.

### 2.3 Cerebral ischemia-reperfusion model

The method for preparing the transient middle cerebral artery occlusion (MCAO) model was as described in Ref. [25]. Briefly, male mice were first anesthetized with 2.5 % isoflurane (RWD Life Science, Shenzhen, China). Subsequently, a midline cervical incision was made to expose the left common carotid artery (CCA), internal carotid artery (ICA), and external carotid artery (ECA). The CCA was ligated with a loose knot, while the ECA was ligated with two monofilaments. One monofilament was placed at the distal end of the ECA, and the other was used to ligate both the ECA and the ICA bifurcation simultaneously. An incision was then made between the two ligatures on the ECA for the insertion procedure. A 4-0 nylon monofilament with a diameter of 0.25 ± 0.03 mm and a silicone-coated tip was carefully inserted into the stump of the ECA and advanced into the ICA to effectively occlude the middle cerebral artery (MCA). A laser speckle contrast imaging system (RFLSI III, RWD Life Science) was used to acquire cerebral blood flow (CBF) images. After 2 h of MCAO, the monofilament was removed to initiate reperfusion. Mice in the sham-operated group underwent the same surgical procedure but without the insertion of the occluder.

For a single experiment,ten C57BL/6 mice were utilized as the sham-operated group (Sham group). Thirty C57BL/6 mice were employed to establish the middle cerebral artery occlusion/reperfusion (MCAO/R) model, with five mice in each group being anesthetized at 24 h, 48 h, and 72 h, respectively. The perilesional brain tissues from each mouse were extracted and stored at −80 °C for subsequent analyses, including Western blot (WB) and quantitative real-time polymerase chain reaction (qPCR). Each experiment was repeated three times (n = 5 per group). Additionally, five mice from each group were anesthetized and sacrificed to obtain paraffin-embedded sections. The paraffin section with the largest area of infarction from each mouse was selected for immunofluorescence staining. A randomly chosen field of view at the same magnification from one section of each mouse was used for quantitative analysis. Mice were scored according to the modified Neurological Severity Score (mNSS) criteria before anesthesia.

Four weeks prior to the induction of the MCAO model, adeno-associated viruses (AAVs) were injected into the hippocampal region of the ischemic penumbra, resulting in 20 mice with PHGDH knockdown (AAV9-GFAP-siPHGDH) and 20 mice with PHGDH overexpression (AAV9-GFAP-PHGDH). Another 40 mice were injected with the corresponding control virus. After 4 weeks of normal housing, stable expression of AAV was achieved.

For a single experiment, after stable expression was achieved, 20 PHGDH-knockdown mice or 20 PHGDH-overexpressing mice, along with their respective 20 control mice, were used to establish the MCAO/R model. At 48 h post-modeling, 8 mice were randomly selected from each group for behavioral tests. Then, all mice were anesthetized and sacrificed. Ischemic brain tissues from five mice in each group were used for WB analyses (n = 5 per group). Brain tissues from five mice in each group were used to obtain paraffin-embedded sections and for immunofluorescence staining (n = 5 per group). Additionally, ten mice in each group were used for survival analysis (n = 10 per group).

For experiments in the chronic phase of mouse MCAO/R, we divided both the PHGDH-knockdown experiment and the PHGDH-overexpression experiment into 4 groups, respectively, based on two variables: the presence or absence of AAV intervention and the presence or absence of MCAO/R modeling. Corresponding behavioral tests and pathological examinations were performed at 14 days after reperfusion.

### 2.4 Cell culture and treatment

Astrocyte C8D1a cells were cultured in high-glucose DMEM (Gibco) supplemented with 10 % fetal bovine serum (FBS, Gibco) and 1 % penicillin-streptomycin (Thermo Fisher Scientific), maintained at 37 °C in a humidified atmosphere with 5 % CO_2_. When cell confluency reached 80–90 %, cells were passaged at a ratio of 1:3 using 0.25 % trypsin-EDTA (Thermo Fisher Scientific) for digestion.

HT-22 cells were cultured in DMEM medium (Gibco) containing 10 % FBS and 1 % penicillin-streptomycin, under the same incubator conditions (37 °C, 5 % CO_2_). Passaging was performed at a ratio of 1:4 using 0.05 % trypsin-EDTA when confluency reached approximately 80 %.

For experiments, cells were seeded into 6-well or 96-well plates with densities adjusted to experimental needs: C8D1a cells at 1 × 10^6^ cells/well and HT-22 cells at 8 × 10^5^ cells/well in 6-well plates; C8D1a cells at 1 × 10^4^ cells/well and HT-22 cells at 8 × 10^3^ cells/well in 96-well plates. After seeding, cells were allowed to adhere for 24 h, followed by synchronization with serum-free medium for 4 h before undergoing treatments such as oxygen-glucose deprivation (OGD) as per experimental design.

Cells were seeded at specific ratios to investigate cell–cell interactions. In the experimental groups, C8D1a cells were seeded at a density of 5 × 10^5^ cells/well in the bottom chambers, and HT-22 cells were seeded at 4 × 10^5^ cells/insert. As controls, single cultures of C8D1a cells (1 × 10^6^ cells/well) and HT-22 cells (8 × 10^5^ cells/insert) were also set up in parallel. After seeding, the co-culture and single-culture systems were incubated at 37 °C with 5 % CO_2_ for 24 h to allow cell attachment. Following attachment, the medium in both the bottom chambers and inserts was replaced with serum-free medium for 4 h to synchronize the cells.

Subsequently, oxygen-glucose deprivation (OGD) treatment was applied to both co-culture and single-culture groups. For OGD, the medium was replaced with glucose-free DMEM pre-gassed with a mixture of 95 % N_2_ and 5 % CO_2_ for 30 min, and then the plates were transferred to an anaerobic chamber (1 % O_2_, 5 % CO_2_, 94 % N_2_) at 37 °C for the specified duration of 2–8 h. After OGD, the cells were returned to normoxic conditions with fresh complete medium to initiate the reperfusion phase. Cell viability, morphological changes, and relevant molecular markers were then analyzed at designated time points post-treatment.

### 2.5 Western blot

Western blotting was performed according to the previously described methods. Astrocytes were collected for protein extraction following different treatments. Mice were anesthetized at various time points after MCAO/R, and the perilesional brain tissues were harvested for Western blot (WB) analysis. The following reagents were used: BCA Protein Assay Kit (Thermo Fisher Scientific, USA), RIPA Lysis Buffer (Thermo Fisher Scientific, USA), Protease Inhibitor Cocktail, Fast Transfer Buffer, and Blocking Buffer (AccuRef Scientific, China). The following antibodies were used: mouse anti-PHGDH (1:5000; Proteintech), rabbit anti-GADMD (1:1000; CST), rabbit anti-GSDMD^Nterm^ (1:2000; Abclonal), rabbit anti-GSDMD (1:2000; Affinity), rabbit anti-AIM2 (1:2000; Proteintech), mouse anti-ASC (1:1000; CST), rabbit anti-Caspase-1 (1:2000; Proteintech), rabbit anti-Cleaved Caspase-1 (1:1000; CST), rabbit anti-IL-1β (1:2000; Abclonal), rabbit anti-IL-18 (1:2000; Abclonal), rabbit anti-β-actin (1:2000; Proteintech),and goat anti-mouse/rabbit secondary antibody (1:10,000; Abcam).

### 2.6 ELISA

Cell supernatants from different treatment groups were collected for ELISA analysis. ELISA was performed strictly in accordance with the manufacturer's instructions. The following ELISA kits were used for detection: Mouse IL-1β ELISA Kit (Elabscience, China) and Mouse IL-18 ELISA Kit (Elabscience, China).

### 2.7 Behavioral experiments in animals

To assess the behavioral changes in animals following cerebral ischemia-reperfusion injury, open-field and rotarod tests were sequentially conducted.

The open-field test was performed in a soundproof room with uniform lighting, using a black square open-field box measuring 50 cm × 50 cm in length and width, and 40 cm in height. The floor of the box was divided into 25 equal square sections. Prior to the test, animals were acclimated to the experimental environment for 30 min. During the test, animals were gently placed in the center of the open-field box, and their activities over a 10-min period were recorded using a camera mounted above. The total distance traveled, average speed, time spent in the center zone, and number of entries into the center zone were automatically analyzed using the ANY-maze behavioral analysis system. After each test, the open-field box was cleaned with 75 % ethanol to eliminate residual odors that might affect subsequent experiments.

The rotarod test was conducted using a rotating rod with a diameter of 3 cm and a non-slip textured surface. Animals underwent a 3-day adaptation training prior to the experiment, with three sessions per day. The starting speed was 4 rpm, gradually accelerating to 40 rpm, with each training session lasting 5 min. During the formal test, animals were placed on the rotating rod, which started at 4 rpm and accelerated continuously at a rate of 1 rpm/s. The latency to fall from the rod was recorded. Each animal was tested three times, with a 10-min interval between tests, and the average value was taken as the final result.

All behavioral experiments were conducted 48 h after the induction of the cerebral ischemia-reperfusion model. The experimenters were blinded to the animal group assignments when performing the tests and recording data.

### 2.8 Immunofluorescence (IF) and TUNEL staining

Immunofluorescence staining was performed as previously described [26]. Mice were anesthetized after MCAO/R, and the brain sections with the largest infarct area were selected for immunofluorescence analysis. The following antibodies were used: mouse anti-GFAP (1:300; CST), mouse anti-Iba1 (1:250; GeneTex), mouse anti-NeuN (1:300; CST), rabbit anti-PHGDH (1:200; Proteintech), rabbit anti-AIM2 (1:200; Proteintech), rabbit anti-Caspase-1 (1:300; Proteintech), rabbit anti-GSDMD (1:200; Proteintech), donkey antirabbit IgG (H + L) highly cross-adsorbed secondary antibody, Alexa Fluor Plus 488 (1:1000; Invitrogen), and donkey anti-mouse IgG (H + L) highly cross-adsorbed secondary antibody, Alexa Fluor Plus 555 (1:1000; Invitrogen).

To detect neuronal apoptosis, TdT-mediated dUTP nick-end labeling (TUNEL) staining was performed with a One-Step TUNEL Assay Kit (Beyotime). Brain slices were first incubated with anti-NeuN primary antibody (1:300, CST) at 4 °C overnight. After washing with PBS, TUNEL staining was carried out in the dark at 37 °C for 1 h according to the kit instructions. To visualize cell nuclei, 4′,6-diamidino-2-phenylindole (DAPI, 1:1000, Sigma) was used for counterstaining. For quantitative analysis, images were captured from four randomly selected regions in each sample. The percentage of TUNEL-positive neurons (green fluorescence) relative to the total number of NeuN-stained neurons (red fluorescence) was calculated using ImageJ software. Each staining experiment was repeated three independent times, and data were presented as the mean ± standard deviation.

### 2.9 ROS and MitoSox staining

For the detection of cellular reactive oxygen species (ROS), we used the Reactive Oxygen Species Assay Kit (red fluorescence; Solarbio, China) and performed the operation strictly in accordance with the manufacturer’ s instructions. The Reactive Oxygen Species Assay Kit is a kit that utilizes fluorescent probes for ROS detection. Specifically, dihydroethidium (DHE) can freely penetrate the cell membrane of living cells to enter the cytoplasm, where it is oxidized by intracellular ROS to form ethidium bromide. Ethidium bromide can intercalate into chromosomal DNA, emitting red fluorescence. The content and changes of ROS in living cells can be determined based on the generation of red fluorescence. After collecting cells at an appropriate density, the stock solution was diluted to a concentration of 10 μM with serum-free medium. The cells were incubated at 37 °C for 30 min, followed by two washes with phosphate-buffered saline (PBS). Finally, the cells were observed under a fluorescence microscope with a consistent exposure time.

For MitoSox staining, the MitoSOX™ Red Mitochondrial Superoxide Indicator (Thermo Fisher Scientific, America) was employed, with all steps carried out according to the kit's instructions. Following staining, the cells were observed and photographed using Live-Cell Analysis System with an excitation wavelength of 396 nm and an emission wavelength of 610 nm.

### 2.10 Mitochondrial membrane potential detection

Mitochondrial membrane potential (ΔΨm) was detected using the Mitochondrial Membrane Potential Assay Kit (Beyotime, China). Astrocytes were seeded into confocal dishes at a density of 20,000 cells per dish. After OGD/R treatment, the cells were rinsed twice with pre-warmed PBS. Subsequently, JC-1 working solution was added, and the cells were incubated for 30 min in a 37 °C incubator with 5 % carbon dioxide (CO_2_). Thereafter, the cells were washed three times with JC-1 staining buffer to remove unbound dye. Then cells were observed using a confocal laser scanning microscope.

### 2.11 Mitochondrial stress test

Seahorse XFp/XF96 Extracellular Flux Analyzer (Agilent Technologies) was used to measure cellular oxygen consumption rate (OCR). XF Base Medium (Agilent) was supplemented with 10 mM d-glucose, 2 mM l-glutamine, and 1 mM sodium pyruvate, adjusted to pH 7.4 with 1 M NaOH, and pre-warmed to 37 °C before use. Astrocytes (2 × 10^4^ cells/well) were seeded into XF cell culture microplates 48 h prior to analysis and subjected to OGD/R treatment. One hour before the assay, the medium was replaced with serum-free medium to eliminate serum interference. For the mitochondrial stress test, 1 μM oligomycin (ATP synthase inhibitor), 1 μM carbonyl cyanide-4-(trifluoromethoxy)phenylhydrazone (FCCP, uncoupler), and 0.5 μM rotenone/antimycin A (complex I/III inhibitors) were loaded into reagent ports A, B, and C of the XF Sensor Cartridge, respectively, which was hydrated with XF Calibrant overnight at 37 °C without CO_2_. The assay was run with a 3-min mix, 2-min wait, and 3-min measure cycle; OCR was recorded continuously for 90 min. Data were normalized to total cellular protein content (measured by BCA assay) and analyzed using Wave Software (Agilent), with parameters including basal respiration, ATP-linked respiration, maximal respiration, and spare respiratory capacity calculated as per standard formulas.

### 2.12 Untargeted metabolomics

Metabolomics analysis was conducted in collaboration with Guangzhou Genedenovo Biotechnology Co., Ltd. (Guangzhou, China), encompassing mass spectrometry (MS) analysis and bioinformatics analysis. Briefly, following LV-shPHGDH transfection and subsequent OGD/R treatment, cells were collected and lysed with 1 mL of extraction solvent (methanol: acetonitrile: water, 2:2:1, v/v). Quality control samples were prepared by mixing equal volumes of all samples from each group. Both the experimental and control groups underwent four biological replicates.

Liquid chromatography-tandem mass spectrometry (LC-MS/MS) analysis was performed using an ultra-high-performance liquid chromatography (UHPLC) system (model 1290, Agilent Technologies, Santa Clara, CA, USA). The raw MS data files were converted to mzML format using ProteoWizard and processed with the R package XCMS (version 3.2). Metabolite variations between samples were calculated based on LC-MS spectra in both positive and negative ion modes. Metabolites with a *P* value less than 0.05 were considered differentially expressed.

For statistical analysis, principal component analysis (PCA), partial least squares-discriminant analysis (PLS-DA), and orthogonal partial least squares-discriminant analysis (OPLS-DA) were conducted using the R software package to compare data among groups.

### 2.13 Transcriptomics analysis

**Fig. 1:**
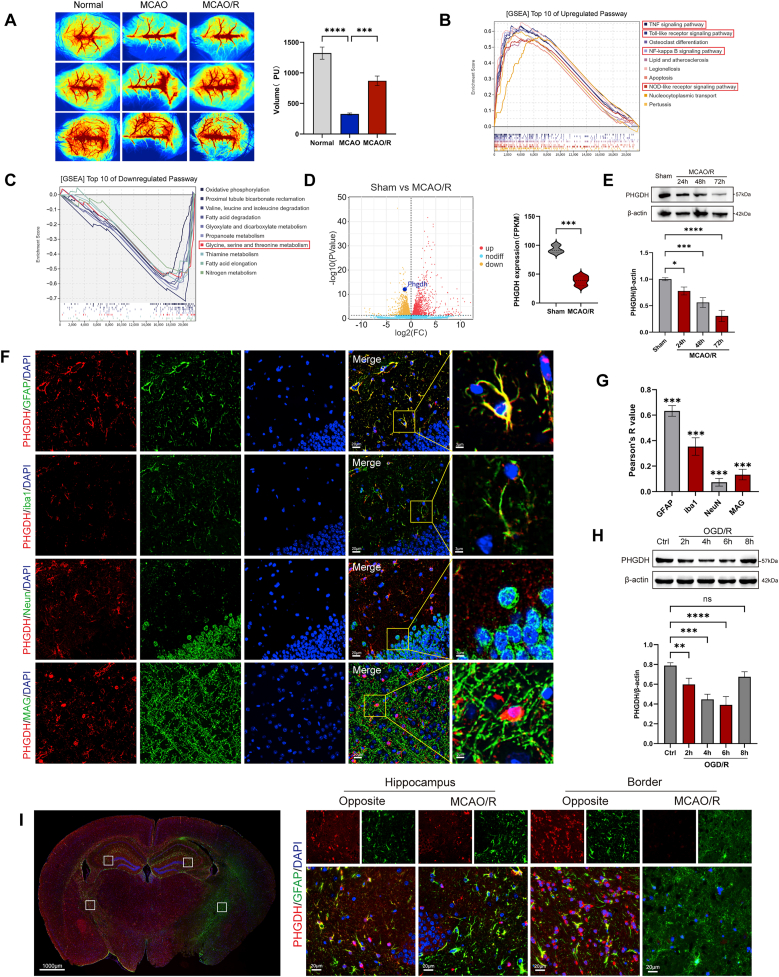
Temporal and spatial expression of PHGDH following cerebral ischemia-reperfusion. (A) Pseudo-colored images and cerebral blood flow statistics obtained using laser speckle flowmetry in mice before and after ischemia and reperfusion. (B, C) KEGG enrichment analysis of transcriptome sequencing in Sham and MCAO/R groups. (D) mRNA expression of PHGDH following MCAO/R. (E) Western blot analysis of PHGDH protein expression at 24 h, 48 h, and 72 h following MCAO/R. (F) Immunofluorescence staining of PHGDH (red) and GFAP, Iba-1, NeuN, MAG (green) in different brain cell types. Scale bar = 20 μm; enlarged scale bar = 3 μm. (G) Colocalization analysis of PHGDH with other cell markers via immunofluorescence staining. (H) Western blot analysis of PHGDH protein expression in cultured astrocytes subjected to OGD/R for 2 h, 4 h, 6 h, and 8 h. (I) Immunofluorescence staining of PHGDH (red) and GFAP (green) in brain sections following MCAO/R. Scale bar = 1000 μm or 20 μm. Statistical analysis was performed using one-way ANOVA followed by post hoc Tukey's test for multiple comparisons. Each experiment was repeated independently at least three times. Values are expressed as mean ± SD. *∗P < 0.05* indicated that the difference between the two groups was statistically significant. *∗P<0.05, ∗∗P<0.01, ∗∗∗P<0.001, ∗∗∗∗P<0.0001*.

Transcriptomics analysis was performed on samples collected from the ischemic penumbra of mice subjected to MCAO/R and compared with the same region from sham-operated mice (Fig. 1). Additionally, transcriptomics analysis was conducted on cells treated with LV-shPHGDH followed by OGD/R treatment (Fig. 3). The entire process was carried out under RNase-free conditions. Total RNA was extracted from the ACC using TRIzol reagent (Thermo Fisher Scientific), strictly following the manufacturer's protocol. For each sample, 1 μg of total RNA was used to construct the sequencing library. Briefly, mRNA was enriched and fragmented using a fragmentation buffer, followed by reverse transcription to generate cDNA using random primers. The cDNA fragments were purified, end-repaired, and polyadenylated (poly(A) tailing) before being ligated to Illumina sequencing adapters. The ligation products were size-selected via agarose gel electrophoresis, amplified by PCR, and sequenced using the Illumina Novaseq 6000 platform (Gene Denovo Biotechnology).

**Fig. 2:**
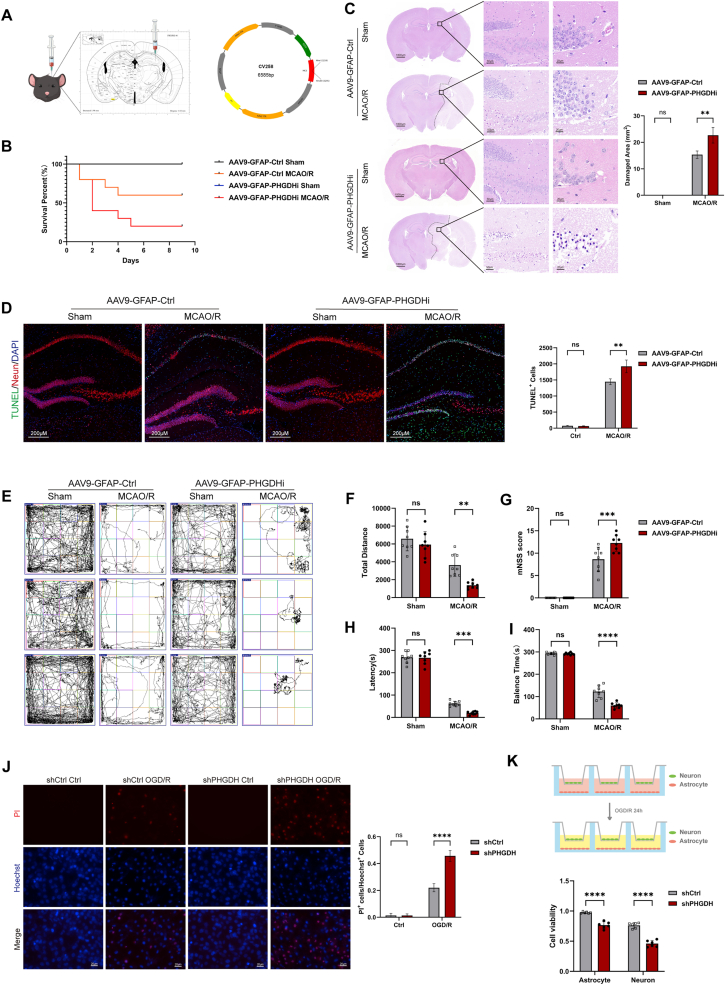
Astrocytic PHGDH knockdown exacerbates cerebral ischemia-reperfusion injury. (A) Schematic diagram of the stereotactic injection site in the brain and the vector construction. (B) Survival analysis of mice after AAV injection and MCAO surgery. (C) Histological analysis of brain neuropathological damage in mice 48 h after MCAO/R using HE staining. Scale bars = 1000 μm or 50 μm. (D) TUNEL staining with Neun co-staining in the hippocampal region of mice brains after MCAO surgery, and quantification of TUNEL-positive cells. Scale bars = 200 μm. (E) Open-field test to assess motor function in mice 48 h after MCAO/R. (F) Quantification of total distance traveled in the open-field test to evaluate motor deficits. (G) mNSS scoring of mice after MCAO/R. (H) Latency analysis in the rotarod test in mice after MCAO/R. (I) Balance time analysis in the rotarod test in mice after MCAO/R. (J) PI (red) and Hoechst (blue) co-staining of cells after OGD/R treatment, with quantification of PI-positive cells. Scale bars = 20 μm. (K) Co-culture of astrocytes and neurons, with cell viability assessed by CCK-8 assay after OGD/R treatment. Statistical analysis was performed using two-way ANOVA. Each experiment was repeated independently at least three times. Values are expressed as mean ± SD. *∗P < 0.05* indicates a statistically significant difference between the two groups. *∗P<0.05, ∗∗P<0.01, ∗∗∗P<0.001, ∗∗∗∗P<0.0001.*

**Fig. 3:**
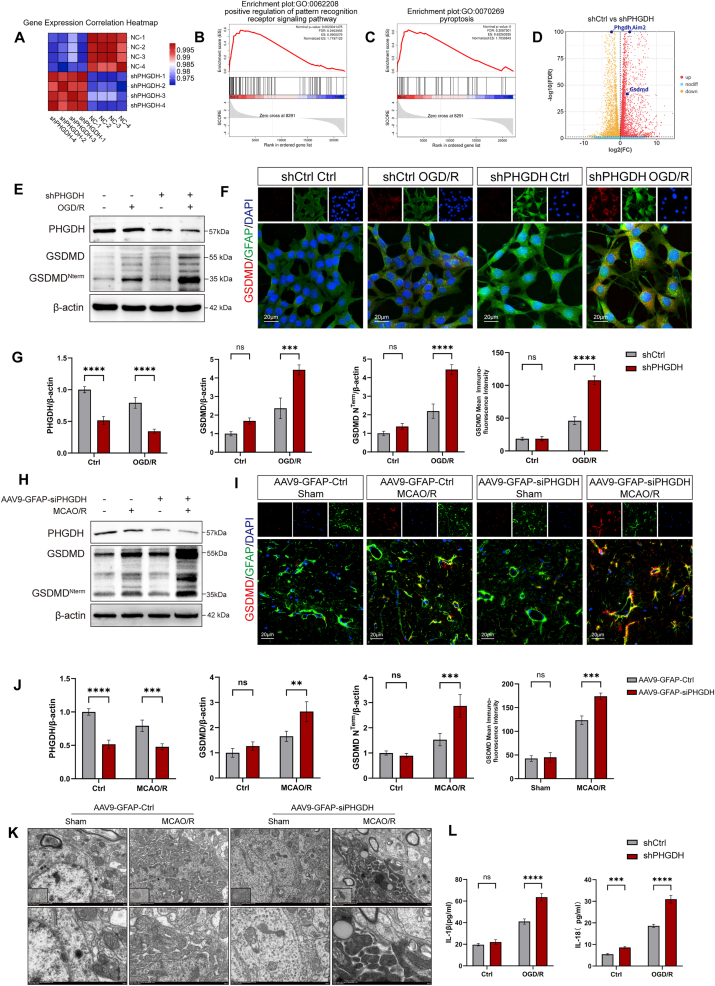
PHGDH knockdown potently induces pyroptosis in astrocytes. (A) Correlation heatmap analysis of RNA sequencing data from astrocytes treated with OGD/R, comparing the shPHGDH group with the shCtrl group. (B, C) RNA sequencing analysis showing upregulation of the pattern recognition receptor signaling pathway and pyroptosis pathway in the shPHGDH group compared to the shCtrl group, with P = 0.0023 or P < 0.0001. (D) Volcano Plot of protein expression in the shPHGDH group compared to the shCtrl group in astrocytes after OGD/R, with red indicating upregulation and yellow indicating downregulation. (E) Western blot analysis of protein expression of PHGDH, GSDMD, and GSDMD^Nterm^ in astrocytes after OGD/R. (F) Immunofluorescence staining of GSDMD (red) and GFAP (green) in astrocytes after OGD/R. Scale bars = 20 μm. (G) Quantitative analysis of protein expression in astrocytes after OGD/R. (H) Western blot analysis of protein expression of PHGDH, GSDMD, and GSDMD^Nterm^ in mouse hippocampal tissue after MCAO/R. (I) Immunofluorescence staining of GSDMD (red) and GFAP (green) in the hippocampal region of mice after MCAO/R. Scale bars = 20 μm. (J) Quantitative analysis of protein expression in the hippocampal region of mice after MCAO/R. (K) Transmission electron microscopy images of astrocytes in the hippocampal region of mice after MCAO/R. Scale bars = 5 μm, 2 μm or 500 nm. (L) ELISA detection of IL-1β and IL-18 secretion in cell culture supernatant. Statistical analysis was performed using two-way ANOVA and Interaction effects were considered significant when P < 0.05. Each experiment was repeated independently at least three times. Values are expressed as mean ± SD. *∗P < 0.05* indicates a statistically significant difference between the two groups. *∗P<0.05, ∗∗P<0.01, ∗∗∗P<0.001, ∗∗∗∗P<0.0001.*

Differentially expressed genes (DEGs) were identified by comparing RNA expression levels between groups. Transcripts with a false discovery rate (FDR) of less than 0.05 and an absolute fold change of ≥1 were considered differentially expressed. Pathway enrichment analysis was performed using the KEGG and GO databases to elucidate the biological functions and pathways associated with the identified DEGs.

### 2.14 Transmission electron microscopy (TEM)

Brain tissue was fixed using an electron microscopy fixative and processed according to the previously described method. Sections were scanned using the H7800 transmission electron microscope (Hitachi, Japan).

### 2.15 Statistical analysis

Statistical analyses were performed using SPSS software (version 17.0). Data are presented as mean ± standard deviation (SD). Each experiment was repeated independently at least three times. Parametric or non-parametric tests were selected based on the homogeneity of variance. Image J software was used to analyze data such as gray value analysis for protein quantification, mean fluorescence intensity, pathological lesion area, and mitochondrial area. Statistical differences were analyzed using Student's t-test, one-way or two-way analysis of variance (ANOVA) depending on the comparison scenario, with Sidak or Tukey's multiple comparisons test applied when appropriate. A p-value less than 0.05 was considered statistically significant.

## 3 Results

### 3.1 PHGDH expression is initially reduced following cerebral ischemia-reperfusion injury

Ischemic stroke elicits both primary and secondary brain injury cascades. A hypoperfused penumbral zone develops around the ischemic core, initiating inflammatory responses and oxidative stress. We successfully generated a mouse MCAO/R model and validated the induction using laser speckle flowmetry imaging (Fig. 1A). In line with prior research, the ischemic penumbra is defined by reactive astrogliosis, manifested as morphological alterations and upregulated GFAP expression (Fig. S1A). RNA sequencing of sham and MCAO/R mice revealed significant upregulation of inflammation-associated pathways, notably the TNF signaling pathway, Toll-like receptor (TLR) signaling pathway, and NOD-like receptor (NLR) signaling pathway (Fig. 1B). Notably, among the downregulated pathways, the glycine, serine, and threonine metabolism was most significantly altered (Fig. 1C), accompanied by a pronounced decrease in PHGDH expression (Fig. 1D). In vivo, serine is converted to glycine and one-carbon units through the catalytic activity of serine hydroxymethyltransferase (SHMT), whereas threonine biosynthesis also depends on serine as a precursor. Therefore, as a rate-limiting enzyme in the de novo serine biosynthesis pathway, PHGDH is critical for maintaining amino acid metabolic homeostasis. Sequencing results were validated via Western blot and IF staining, which demonstrated that PHGDH expression in the ischemic penumbra of MCAO mice was persistently suppressed during the 72-h acute phase (Fig. 1E). Co-immunofluorescence staining of primary brain cell types indicated that PHGDH exhibited the highest expression levels in astrocytes and was nearly undetectable in neurons (Fig. 1F and G). Considering that astrocytes are the most abundant glial cell population in the brain, functionally capable of both initiating and responding to neuroinflammation in neurodegenerative contexts, astrocytes were chosen as the focal cell type for this study. IF staining revealed that, when compared to the contralateral hemisphere, PHGDH expression was significantly decreased in astrocytes at the cortical border and hippocampal region of the ischemic penumbra after MCAO (Fig. 1I). In line with in vivo results, in vitro OGD/R experiments in astrocytic C8D1a cells confirmed a significant reduction in PHGDH expression via Western blot analysis (Fig. 1H). Notably, PHGDH expression reached its lowest point at 6 h post-OGD, subsequently undergoing a modest recovery. In summary, both in vivo and in vitro experiments have demonstrated that PHGDH expression is downregulated following I/R, concomitant with the activation of inflammatory pathways. Therefore, we continued to investigate whether there is a relationship between these two phenomena.

### 3.2 Astrocytic PHGDH knockdown exacerbates cerebral ischemia-reperfusion injury

We utilized AAV9-GFAP-siPHGDH to achieve astrocyte-specific knockdown of PHGDH expression in the mouse brain. Four weeks before MCAO surgery, AAV9-GFAP-siPHGDH and AAV9-GFAP-Ctrl were delivered by stereotaxic injection into the hippocampal formation (Fig. 2A). Knockdown efficiency was validated by IF staining and qRT-PCR analyses (Fig. S1B and C). Subsequently, mice underwent MCAO surgery to induce ischemic stroke, followed by 48 h of reperfusion. Notably, AAV9-GFAP-siPHGDH transduction significantly affected post-MCAO/R mouse survival, leading to a substantial reduction in survival rate (Fig. 2B). Compared to the AAV9-GFAP-Ctrl group, HE staining showed that, AAV9-GFAP-siPHGDH alone did not induce brain tissue injury. However, following MCAO/R modeling, the hippocampal formation displayed reduced eosinophilic staining, cytoplasmic component dissolution, neuronal nuclear pyknosis, and cytoplasmic vacuolization. In contrast, hippocampal injury in the AAV9-GFAP-siPHGDH group was significantly exacerbated, with expanded lesion scope, culminating in liquefactive necrosis formation (Fig. 2C). TUNEL staining further confirmed a substantial increase in TUNEL-positive cells in the AAV9-GFAP-siPHGDH group (Fig. 2D).

Subsequently, a series of behavioral assays were performed to evaluate neurological function. The open-field test was used to assess the effect of astrocyte-specific PHGDH knockdown on murine locomotor function. Results showed no significant difference in ambulatory distance among sham-operated groups. However, MCAO surgery significantly impaired murine locomotor function, as evidenced by a marked reduction in ambulatory distance in the AAV9-GFAP-siPHGDH group compared to the AAV9-GFAP-Ctrl group (Fig. 2E and F). Neurosensory and motor functions were further evaluated using the mNSS Score at 48 h post-MCAO/R. Results revealed significantly higher mNSS scores in the AAV9-GFAP-siPHGDH group post-MCAO (Fig. 2G), indicating that astrocyte-specific PHGDH knockdown exacerbated sensorimotor function impairment after stroke.

Motor balance function was additionally assessed using the rotarod test in post-stroke mice. Results showed that the AAV9-GFAP-siPHGDH group displayed significantly impaired rotarod performance post-MCAO, characterized by reduced latency to fall (Fig. 2H) and shortened balance duration (Fig. 2I).

Lentivirus-mediated shRNA was also used to deplete PHGDH expression in astrocytes in vitro (Fig. S1D). After OGD/R treatment, PI staining demonstrated a marked increase in cell death in the shPHGDH group (Fig. 2J). To model astrocyte-neuron crosstalk post-stroke in vitro, astrocytes were co-cultured with HT-22 neurons. Results demonstrated that following 24 h of OGD/R, astrocyte viability in the shPHGDH group was reduced by 20.87 ± 2.35 %, accompanied by a significant 30.29 ± 2.89 % decrease in neuronal viability (Fig. 2K).

Given that I/R injury is persistent, we further investigated the effect of low PHGDH expression on the long-term complications of I/R injury. We found that the expression of PHGDH remained persistently low from day 7 to day 21 after I/R (Fig. S3D). Pathological examination results showed that the degree of hippocampal neuronal pyknosis in the AAV-GFAP-siPHGDH group was significantly higher than that in the AAV9-GFAP-Ctrl group after I/R (Fig. S3C). Behavioral test results revealed the following: in the open field test, the total movement distance of the AAV9-GFAP-siPHGDH group was significantly shorter than that of the AAV9-GFAP-siCtrl group after I/R (Fig. S3E); in the rotarod test, the latency to fall and balance time of the AAV9-GFAP-siPHGDH group were significantly lower than those of the AAV9-GFAP-siCtrl group after I/R; and consistent results were obtained for neurological function scores (Fig. S3F).

Therefore, we concluded that PHGDH downregulation is not transient—PHGDH levels remain persistently reduced during the chronic recovery phase. Moreover, astrocyte-specific PHGDH knockdown also exerts a significant impact on the long-term complications of mice after I/R.

Collectively, these results indicate that astrocytic PHGDH knockdown exacerbates neuronal injury following I/R, amplifying both pathological damage and functional deficits.

### 3.3 PHGDH knockdown potently induces pyroptosis in astrocytes

RNA sequencing and metabolomics analyses were further performed on shCtrl and shPHGDH groups after OGD/R treatment. Correlation heatmap analysis and hierarchical clustering indicated that shCtrl and shPHGDH groups were clearly segregated into distinct metabolic clusters (Fig. 3A). A total of 4533 DEGs were identified, including 2043 upregulated and 2490 downregulated genes (Fig. S1E). Notably, while inflammation-associated pathways such as TNF signaling, TLR signaling, and NLR signaling pathways were significantly upregulated post-I/R, with marked induction of GSDMD expression, KEGG and GO enrichment analyses demonstrated that these inflammatory pathways were further upregulated in the shPHGDH group relative to the shCtrl group after OGD/R (Fig. S1F–H). Conversely, serine synthesis and metabolism pathways were significantly suppressed (Fig. S1I and G). Notably, the pattern recognition receptor signaling and pyroptosis pathways were markedly upregulated (Fig. 3B and C), and PHGDH knockdown was associated with a several-fold increase in AIM2 and GSDMD expression (Fig. 3D). GSDMD expression was evaluated in vivo and in vitro via WB and IF analyses. In vitro studies demonstrated that, when compared to the shCtrl group, GSDMD and its N-terminal fragment expression were significantly upregulated in PHGDH-knockdown astrocytes after OGD/R (Fig. 3E and G). IF staining revealed GSDMD-GFAP colocalization, with significantly elevated GSDMD expression in the shPHGDH group compared to the shCtrl group following OGD/R (Fig. 3F and G).

AAV9-GFAP-siPHGDH was further utilized to achieve astrocyte-specific PHGDH knockdown in vivo. After MCAO/R, hippocampal tissue was harvested for WB and IF analyses. Results similarly showed that astrocytic PHGDH knockdown resulted in significant upregulation of GSDMD and GSDMD^Nterm^ expression (Fig. 3H–J). Notably, PHGDH knockdown in the sham-operated group did not affect GSDMD or GSDMD^Nterm^ expression.

Upon induction of astrocytic pyroptosis, GSDMD^Nterm^ oligomerizes to form pores in the plasma membrane, thereby causing cellular swelling, osmotic lysis, and release of pro-inflammatory cytokines such as IL-1β and IL-18. Electron microscopy was used to examine pyroptotic pore formation in astrocyte plasma membranes of the AAV9-GFAP-siPHGDH group. After I/R, compared to the AAV9-GFAP-Ctrl group, the AAV9-GFAP-siPHGDH group exhibited higher frequency of pyroptotic pore formation and greater electron density in astrocytes (Fig. 3K), indicating that PHGDH knockdown exacerbates astrocytic pyroptosis after I/R.

Cell supernatants were collected for ELISA-based quantification of IL-1β and IL-18 secretion. After OGD/R treatment, both cytokines were significantly elevated in the shPHGDH group (Fig. 3L). Collectively, these findings indicate that astrocytic PHGDH knockdown potently induces pyroptosis after I/R and enhances pro-inflammatory cytokine release.

### 3.4 PHGDH knockdown potently induces AIM2 inflammasome activation in both in vivo and in vitro models

Pyroptosis is predominantly initiated via the canonical inflammasome pathway, during which AIM2 is activated to assemble the AIM2-ASC-Caspase-1 inflammasome complex [27]. This complex mediates the canonical pathway to proteolytically cleave GSDMD into its N-terminal fragment, which then undergoes oligomerization to form pyroptotic pores, ultimately causing cell pyroptosis [28]. Concurrently, inflammasome activation triggers the proteolytic maturation of pro-IL-1β and pro-IL-18 into their active forms, IL-1β and IL-18, which are subsequently released to propagate neuroinflammation in the central nervous system.

**Fig. 4:**
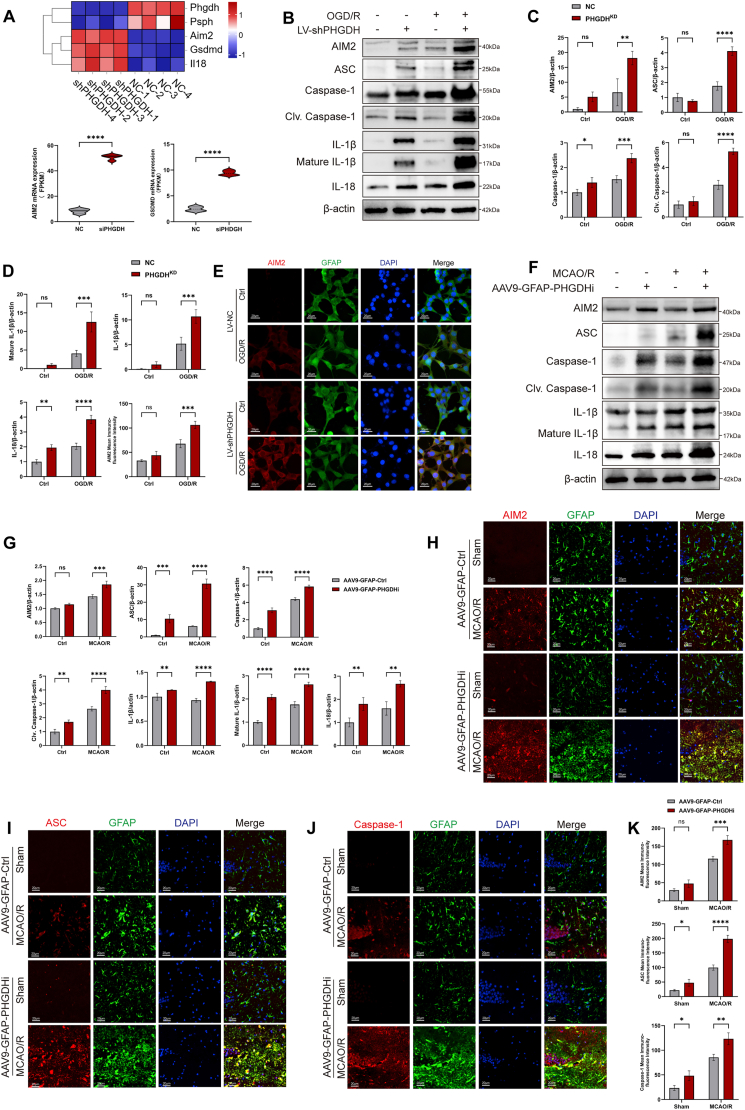
PHGDH knockdown potently induces AIM2 inflammasome activation in both in vivo and in vitro models. (A) Heatmap from RNA sequencing showing mRNA expression levels of AIM2, GSDMD, and other related genes. (B) Western blot analysis of protein expression of AIM2, ASC, Caspase-1, Cleaved Caspase-1, IL-1β, mature IL-1β, IL-18, and β-actin in astrocytes after OGD/R. (C, D) Quantitative analysis of protein expression of AIM2, ASC, Caspase-1, Cleaved Caspase-1, IL-1β, mature IL-1β, IL-18, and β-actin in astrocytes after OGD/R, and statistic of mean immunofluorescence intensity for AIM2 immunofluorescence staining. (E) Immunofluorescence staining of GSDMD (red) and GFAP (green) in astrocytes after OGD/R. Scale bars = 20 μm. (F) Western blot analysis of protein expression of AIM2, ASC, Caspase-1, Cleaved Caspase-1, IL-1β, mature IL-1β, IL-18, and β-actin in the hippocampal region of mice after MCAO/R. (G) Quantitative analysis of protein expression of AIM2, ASC, Caspase-1, Cleaved Caspase-1, IL-1β, mature IL-1β, IL-18, and β-actin in the hippocampal region of mice after MCAO/R. (H–J) Immunofluorescence staining of AIM2, ASC, Caspase-1 (red) and GFAP (green) in the hippocampal region of mice after MCAO/R. Scale bars = 20 μm. (K) Statistics of mean immunofluorescence intensity for AIM2, ASC, and Caspase-1 immunofluorescence staining. Statistical analysis was performed using two-way ANOVA and Interaction effects were considered significant when P < 0.05. Each experiment was repeated independently at least three times. Values are expressed as mean ± SD. *∗P < 0.05* indicates a statistically significant difference between the two groups. *∗P<0.05, ∗∗P<0.01, ∗∗∗P<0.001, ∗∗∗∗P<0.0001.*

In vitro RNA sequencing analysis revealed a striking upregulation of AIM2 expression in PHGDH-knockdown astrocytes (Fig. 4A). These results were subsequently validated by WB and IF analyses. Results demonstrated that after OGD/R treatment, key proteins in the AIM2 inflammasome pathway, including AIM2, ASC, Caspase-1, Cleaved Caspase-1, IL-1β, mature IL-1β, and IL-18, were significantly upregulated in astrocytes, with markedly higher expression in the SHPHGDH group compared to the shCtrl group (Fig. 4B–D). IF staining revealed AIM2-GFAP colocalization and significantly increased AIM2 expression in the shPHGDH group following OGD/R (Fig. 4E).

Consistent results were observed in vivo. After I/R, AIM2 inflammasome-associated proteins were significantly upregulated in the AAV9-GFAP-siPHGDH group (Fig. 4F and G). IF staining revealed colocalization of AIM2, ASC, and Caspase-1 with GFAP, with pronounced upregulation of these proteins following PHGDH knockdown (Fig. 4H–K). Collectively, these results indicate that astrocytic PHGDH knockdown potently activates the AIM2-ASC-Caspase-1 inflammasome following I/R.

**Fig. 5:**
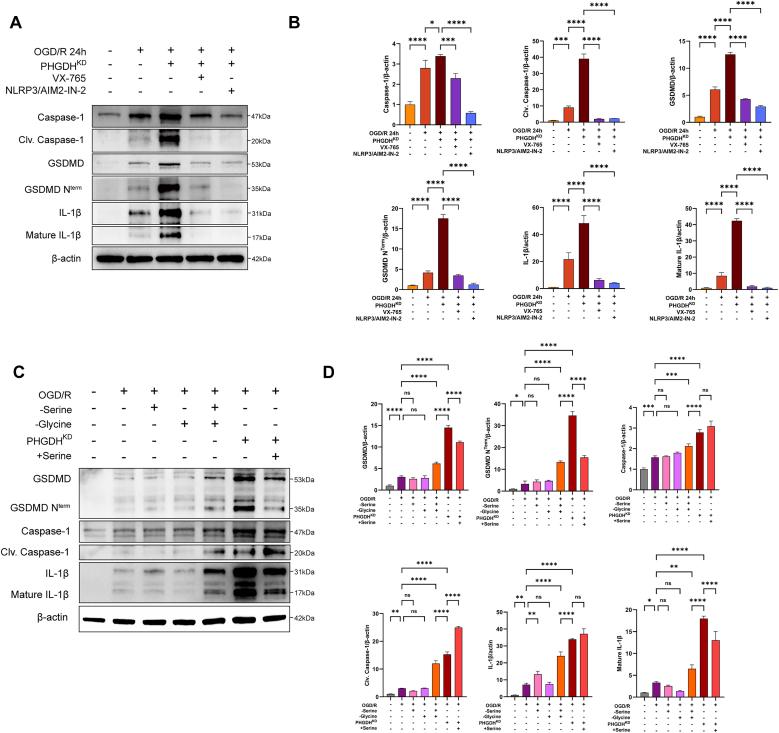
Effects of exogenous inhibitors or serine/glycine on astrocyte pyroptosis. (A, B) Western blot analysis showing the inhibitory effects of exogenously added NLRP3/AIM2-IN-2 or VX-765 on pyroptosis in astrocytes. (C, D) Western blot analysis showing the effects of serine and glycine on pyroptosis in astrocytes. Statistical analysis was performed using one-way ANOVA. Each experiment was repeated independently at least three times. Values are expressed as mean ± SD. *∗P < 0.05* indicates a statistically significant difference between the two groups. *∗P<0.05, ∗∗P<0.01, ∗∗∗P<0.001, ∗∗∗P<0.0001.*

NLRP3/AIM2-IN-2 is a selective inhibitor that disrupts the interaction between AIM2 and the adaptor protein ASC, thereby preventing ASC oligomerization. VX-765 is a Caspase-1-selective inhibitor that blocks Caspase-1 activation. Western blot analysis showed that pre-treatment with these inhibitors before OGD significantly attenuated PHGDH knockdown-induced pyroptosis following OGD/R in astrocytes (Fig. 5A and B). Thus, PHGDH knockdown promotes astrocytic pyroptosis by inducing AIM2 inflammasome activation.

### 3.5 The catalytic function of PHGDH is a key factor inhibiting astrocyte pyroptosis after ischemia-reperfusion

As PHGDH serves as the rate-limiting enzyme in de novo serine biosynthesis, metabolomics analyses revealed a profound reduction in intracellular serine levels in PHGDH-depleted astrocytes, whereas transcriptomics demonstrated significant downregulation of serine metabolic pathways. Consequently, we sought to determine whether serine deficiency mediates PHGDH knockdown-induced pyroptosis in astrocytes. Contrary to expectations, exogenous serine supplementation failed to significantly inhibit GSDMD^Nterm^ expression and other pyroptosis-related factors in PHGDH-depleted astrocytes. Astrocytes cultured in serine-deficient medium exhibited that serine deficiency alone was insufficient to exacerbate pyroptosis after OGD/R treatment. Given the reversible conversion of serine and glycine via SHMT1/SHMT2 in vivo, glycine deficiency alone similarly failed to significantly enhance astrocytic pyroptosis. Therefore, we performed astrocyte culture in medium deficient in both serine and glycine. Results demonstrated that combined serine and glycine deficiency potentiated OGD/R-induced astrocytic pyroptosis, as evidenced by significant upregulation of GSDMD^Nterm^, Caspase-1, and IL-1β (Fig. 5C and D). Notably, single deficiency of serine or glycine alone was insufficient to recapitulate PHGDH knockdown-induced astrocytic pyroptosis. l-phenylalanine, a competitive inhibitor of serine transporters, reduces cellular serine uptake. Western blot analysis indicated that l-phenylalanine supplementation failed to enhance OGD/R-induced astrocytic pyroptosis (Fig. S2A and B). Furthermore, exogenous serine supplementation in PHGDH-depleted astrocytes failed to fully rescue cellular viability.

We further examined the effects of I/R and PHGDH knockdown on PHGDH catalytic activity. The results showed that PHGDH activity was significantly decreased after I/R in both in vivo and in vitro experiments, and PHGDH knockdown also reduced PHGDH catalytic activity. To rule out the influence of PHGDH enzyme content on activity detection, we compared the specific activity of PHGDH by combining it with protein quantification. Consistent with the above findings, the specific activity of PHGDH was also decreased after I/R and PHGDH knockdown (Fig. S3A and B).

Then, we constructed PHGDH mutants, namely NM_016966.3(T57/78A) and NM_016966.3(R135W/V261 M), via site-directed mutagenesis [24]. For PHGDH NM_016966.3(T57/78A), the mutation was targeted at Thr57, a key binding site for glycerate-3-phosphate. This mutation abolished glycerate-3-phosphate binding ability, impaired catalytic activity, and rendered the enzyme in a “pseudo-starvation” state. In contrast, NM_016966.3(R135W/V261 M) inhibited the binding of PHGDH to NAD, reducing its activity in catalyzing the conversion of glycerate-3-phosphate to hydroxypyruvate-3-phosphate, while retaining the ability to bind glycerate-3-phosphate; thus, the enzyme was in a “pseudo-satiation” state. Results showed that both mutants (NM_016966.3(T57/78A) and NM_016966.3(R135W/V261 M)) promoted pyroptosis of astrocytes after OGD/R (Fig. S2B and C). This indicates that low PHGDH expression accelerates the process of astrocyte injury after I/R by inhibiting the catalytic function of PHGDH.

Collectively, these data indicate that serine depletion is a crucial factor underlying astrocyte pyroptosis induced by low PHGDH expression after I/R, and the catalytic function of PHGDH is key to suppressing the exacerbation of astrocyte pyroptosis after I/R.

### 3.6 PHGDH knockdown exacerbates I/R-induced oxidative stress by reducing the production of reduced substances

**Fig. 6:**
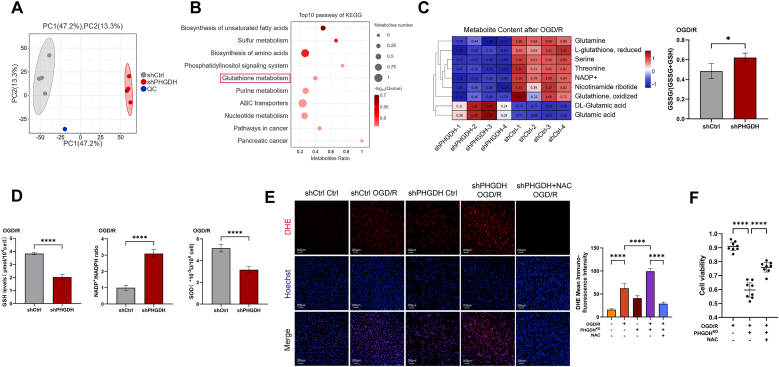
PHGDH knockdown exacerbates I/R-induced oxidative stress by reducing the production of reduced substances. (A) Quality control PCA for the untargeted metabolomics of OGD/R-treated astrocytes was conducted to compare the shPHGDH group with the shCtrl group. (B) KEGG enrichment bubble plot from metabolomics analysis. (C) Heatmap of metabolites related to oxidative stress and bar chart of the GSSG/(GSSG + GSH) ratio. (D) Measurement of GSH levels, SOD activity, and NADP+/NADPH ratio in astrocytes after OGD/R using assay kits. (E) DHE (red) staining to measure rate of cellular ROS production in astrocytes after OGD/R, with and without NAC treatment. Scale bars = 200 μm. (F) CCK-8 assay to assess cell viability in the shCtrl, shPHGDH, and shPHGDH + NAC groups after OGD/R. Statistical analysis was performed using one-way ANOVA or *t*-test. Each experiment was repeated independently at least three times. Values are expressed as mean ± SD. *∗P < 0.05* indicates a statistically significant difference between the two groups. *∗P<0.05, ∗∗P<0.01, ∗∗∗P<0.001, ∗∗∗P<0.0001.*

Untargeted metabolomics analysis was performed on the shCtrl and shPHGDH groups after OGD/R treatment. Using partial least squares discriminant analysis (PLS-DA), the score plot of Principal Component Analysis (PCA) showed a clear separation between the shCtrl group and the shPHGDH group, with good homogeneity within each group (Fig. 6A). A total of 456 differential metabolites were identified, 343 of which were downregulated (Fig. S2D), including a significant reduction in serine levels. In comparison to the shCtrl group, redox homeostasis was severely disrupted in the shPHGDH group, as evidenced by significant alterations in redox-related metabolites. Specifically, glutathione metabolism pathway was significantly enriched (Fig. 6B). Levels of reduced l-glutathione (GSH), glutamine, NADP^+^, and nicotinamide ribotide were significantly decreased, whereas the GSSG/(GSH + GSSG) ratio was significantly elevated (Fig. 6C and S2E), indicating cellular oxidative stress. Quantification confirmed that after OGD/R, the shPHGDH group exhibited significantly lower GSH levels and SOD activity than the shCtrl group, whereas the NADP^+^/NADPH ratio was significantly increased (Fig. 6D). Subsequently, ROS production rate in astrocytes were detected using the DHE probe. In both the Ctrl and OGD/R groups, PHGDH knockdown significantly elevated ROS production rate, whereas exogenous NAC supplementation significantly suppressed ROS generation (Fig. 6E) and rescued pyroptotic astrocytes (Fig. 6F).

### 3.7 PHGDH knockdown induces electron transport chain damage and exacerbates oxidative stress

We further investigated the additional mechanisms by which PHGDH knockdown affects ROS production and astrocyte pyroptosis.

**Fig. 7:**
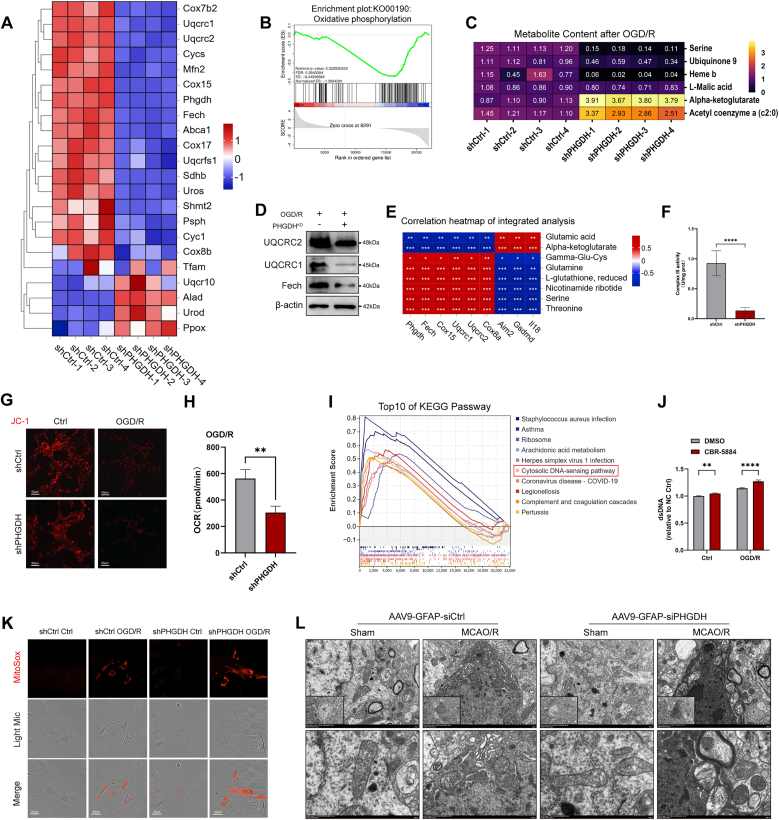
PHGDH knockdown induces electron transport chain damage and exacerbates oxidative stress. (A) Heatmap of gene expression related to the mitochondrial oxidative respiratory chain from RNA sequencing after OGD/R. (B) GSEA analysis reveals significant downregulation of the oxidative phosphorylation pathway. (C) Levels of metabolites related to mitochondrial complex III in astrocytes after OGD/R. (D) Quantitative analysis of key molecular proteins related to mitochondrial complex III function. (E) Integrated analysis heatmap showing correlations between RNA sequencing and metabolomics data after OGD/R. (F) Activity assay of mitochondrial complex III in astrocytes after OGD/R. (G) Measurement of cellular mitochondrial membrane potential. Scale bars = 50 μm. (H) Mitochondrial stress test for detecting dynamic changes in cellular oxygen consumption rate (OCR). (I) Top 10 upregulated pathways from KEGG enrichment analysis after OGD/R. (J) Measurement of dsDNA levels following inhibition of PHGDH activity. (K) MitoSox (red) staining to measure rate of mitochondrial ROS production in astrocytes after OGD/R. Scale bars = 20 μm. (L) Transmission electron microscopy images of mitochondrial morphology in astrocytes in the hippocampal region of mice after MCAO/R. Scale bars = 5 μm, 2 μm and 500 nm. Statistical analysis was performed using two-way ANOVA or *t*-test. Each experiment was repeated independently at least three times. Values are expressed as mean ± SD. *∗P < 0.05* indicates a statistically significant difference between the two groups. *∗P<0.05, ∗∗P<0.01, ∗∗∗P<0.001, ∗∗∗P<0.0001.*

Transcriptomic analysis revealed that genes related to the mitochondrial oxidative respiratory chain were generally downregulated in PHGDH-knockdown astrocytes (Fig. 7A). GSEA analysis showed that after PHGDH knockdown, while genes involved in the serine family amino acid synthesis and metabolism pathways were collectively and significantly downregulated (Fig. S1I and J), the oxidative phosphorylation pathway was also significantly downregulated (Fig. 7B). Metabolomic results demonstrated that PHGDH knockdown reduced serine levels by more than 80 %, accompanied by a significant downregulation of ubiquinone-9 and heme—with heme almost undetectable (Fig. 7C). Ubiquinone-9 is a core carrier of the electron transport chain (ETC), responsible for transferring electrons from mitochondrial Complexes I (NADH-ubiquinone oxidoreductase) and II (succinate-ubiquinone oxidoreductase) to Complex III (ubiquinol-cytochrome c oxidoreductase). Heme serves as a core prosthetic group for mitochondrial ETC Complex III and Complex IV (cytochrome *c* oxidase), and defects in its synthesis directly lead to mitochondrial dysfunction.

Through integrated analysis of transcriptomic and metabolomic data, we found that after OGD/R treatment, concurrent with PHGDH downregulation, genes related to the de novo serine synthesis pathway were collectively downregulated—including PSPH and SHMT2. Additionally, genes encoding key rate-limiting enzymes for heme synthesis (Fech, Uros), as well as genes involved in the synthesis of key components of mitochondrial Complex III (Uqcrc1, Uqcrc2, Uqcrfs1) and multiple genes of the Cox family, were significantly downregulated (Fig. 7A and D). Meanwhile, the expression levels of PHGDH, Fech, Cox15, Cox 8a, Uqcrc1, and Uqcrc2 were positively correlated with the levels of serine, glutathione (GSH), nicotinamide ribotide (the precursor of NAD^+^), and other reduced metabolites, while the levels of pyroptosis-related markers were negatively correlated with the levels of these reduced metabolites (Fig. 7E).

Subsequently, we detected mitochondrial Complex III activity, and the results showed that compared with the control group, PHGDH knockdown significantly reduced its activity (Fig. 7F). We further examined mitochondrial membrane potential and mitochondrial function. JC-1 staining revealed that the mitochondrial membrane potential in the shPHGDH group was significantly lower than that in the shCtrl group after I/R (Fig. 7G). Mitochondrial stress tests showed that the oxygen consumption rate (OCR) of astrocytes in the shPHGDH group was significantly lower than that in the control group after I/R (Fig. 7H and S2F)). This indicates that PHGDH knockdown significantly induces mitochondrial dysfunction after I/R.

Analysis of GSEA enrichment results between the shPHGDH and shCtrl groups after I/R indicated that the cytosolic DNA-sensing pathway was significantly upregulated and ranked among the top 10 upregulated pathways (Fig. 7I). Detection results also showed that inhibition of PHGDH activity significantly increased dsDNA levels (Fig. 7J), suggesting that a large amount of mitochondrial DNA (mtDNA) was released into the cytoplasm, activating the cytosolic DNA-sensing pathway. Notably, dsDNA is a potent activator of the AIM2 inflammasome pathway. We simultaneously measured the mitochondrial ROS production rate and observed mitochondrial morphology under TEM. Consistent with the aforementioned results, PHGDH knockdown impaired the integrity of the ETC and significantly induced mitochondrial oxidative stress (Fig. 7K and S2G). Inhibition of PHGDH enzymatic activity via PHGDH site-directed mutagenesis (T57/78A and R135W/V261 M) also exacerbates mitochondrial oxidative stress (Fig. S2H). Under TEM, astrocytes with PHGDH knockdown exhibited obviously abnormal mitochondrial morphology and blurred mitochondrial cristae compared with the control group, indicating mitochondrial damage (Fig. 7L and S2I).

In conclusion, PHGDH knockdown in astrocytes can significantly impair the integrity of the mitochondrial ETC after I/R, induce mitochondrial oxidative stress, promote the release of mtDNA into the cytoplasm to activate the cytosolic DNA-sensing pathway, and further activate the AIM2 inflammasome pathway, ultimately inducing astrocyte pyroptosis.

### 3.8 PHGDH overexpression attenuates astrocyte pyroptosis and neurological impairment after ischemia-reperfusion

To investigate the effect of PHGDH on ischemia-reperfusion injury after I/R, we conducted in vivo and in vitro experiments using PHGDH-overexpressing adeno-associated virus (AAV-GFAP-PHGDH) and lentivirus (LV-PHGDH^OE^).

**Fig. 8:**
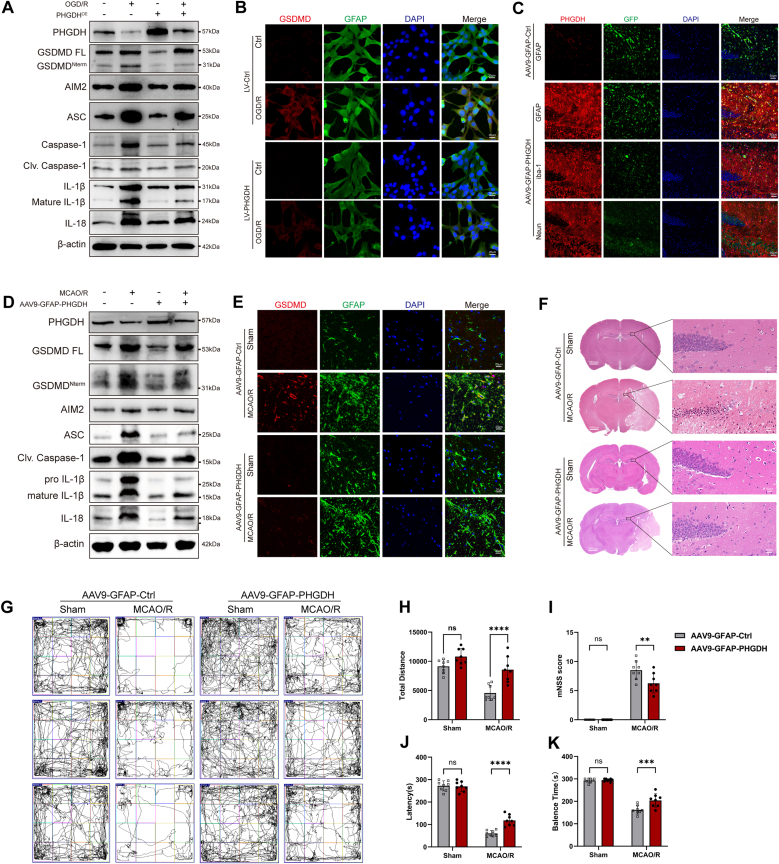
PHGDH overexpression attenuates astrocyte pyroptosis and neurological impairment after ischemia-reperfusion. (A) Western blot analysis of protein expression of PHGDH and pyroptosis marker in astrocytes after OGD/R. (B) Immunofluorescence staining of GSDMD (red) and GFAP (green) in astrocytes after OGD/R. Scale bars = 20 μm. (C) Immunofluorescence staining of astrocyte-specific PHGDH overexpression in astrocytes, microglia, and neurons in the hippocampal region of mice. Scale bars = 50 μm. (D) Western blot analysis of protein expression of PHGDH and pyroptosis marker in mouse hippocampal tissue after MCAO/R. (E) Immunofluorescence staining of GSDMD (red) and GFAP (green) in the hippocampal region of mice after MCAO/R. Scale bars = 20 μm. (F) Histological analysis of brain neuropathological damage in mice after MCAO/R using HE staining. Scale bars = 1000 μm or 50 μm. (G) Open-field test to assess motor function in mice after MCAO/R. (H) Quantification of total distance traveled in the open-field test to evaluate motor deficits. (I) mNSS scoring of mice after MCAO/R. (J) Latency analysis in the rotarod test in mice after MCAO/R. (K) Balance time analysis in the rotarod test in mice after MCAO/R. Statistical analysis was performed using two-way ANOVA and Interaction effects were considered significant when P < 0.05. Each experiment was repeated independently at least three times. Values are expressed as mean ± SD. *∗P < 0.05* indicates a statistically significant difference between the two groups. *∗P<0.05, ∗∗P<0.01, ∗∗∗P<0.001, ∗∗∗∗P<0.0001*.

First, in vitro experiments were carried out, where lentivirus was employed to infect astrocytes and construct a PHGDH-overexpressing model. After stable expression was achieved, the cells were subjected to OGD/R treatment, and a series of pyroptosis-related indicators were detected (Fig. 8A and B and S4A). The results showed that PHGDH overexpression significantly reduced the level of GSDMD^Nterm^ after I/R; the expression of AIM2 inflammasome-related markers was significantly decreased, as were the expressions of IL-1β and IL-18. Notably, PHGDH overexpression in the control group did not upregulate the levels of pyroptosis markers.

Subsequently, AAV9-GFAP-PHGDH was used to achieve astrocyte-specific overexpression of PHGDH in the mouse brain. Four weeks after viral infection, immunofluorescence staining revealed that PHGDH was significantly overexpressed in the hippocampal region, with exclusive expression in astrocytes and negligible expression in neurons and microglia (Fig. 8C). The mice were then subjected to MCAO/R modeling, and the expression of pyroptosis-related markers in hippocampal tissues was detected. Consistent with the in vitro results, PHGDH overexpression significantly reduced GSDMD^Nterm^ levels, downregulated the expression of AIM2 inflammasome-related markers, and decreased the expressions of IL-1β and IL-18 (Fig. 8D, E and S4B). Meanwhile, brain tissue sections were prepared and stained with HE staining. The infarct area in the AAV9-GFAP-PHGDH group after MCAO/R was significantly smaller than that in the control group (Fig. 8F and S4C).

Prior to pathological and molecular biological analyses, behavioral tests were performed on the mice. The open field test showed that the total movement distance of mice in the PHGDH-overexpression group was significantly higher than that in the control group after MCAO/R (Fig. 8G and H). The mNSS indicated that the neurological function score of the PHGDH-overexpression group was lower than that of the control group (Fig. 8I). The rotarod test demonstrated that the motor balance ability of mice in the PHGDH-overexpression group was superior to that in the control group (Fig. 8J and K). Collectively, these results suggested that the recovery of motor function in PHGDH-overexpressing mice after MCAO/R was better than that in the control group.

In conclusion, PHGDH overexpression did not induce pathological damage or neurological impairment, or promote inflammation. Nevertheless, after I/R, overexpression of PHGDH exerted a prominent neuroprotective effect on neurological function, which in turn alleviated ischemia/reperfusion-induced damage and attenuated the development of inflammation.

## 4 Discussion

**Fig. 9:**
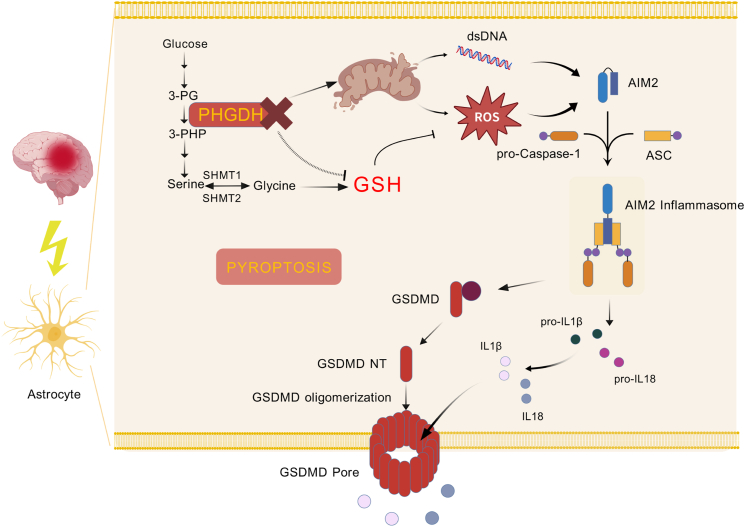
PHGDH knockdown disrupting mitochondrial homeostasis and promoting oxidative stress to exacerbate astrocyte pyroptosis following cerebral ischemia-reperfusion. Created with BioGDP.com.

Ischemic stroke, the most common type of cerebrovascular disease and a leading cause of permanent disability worldwide, relies on revascularization as the primary treatment, yet the mechanisms behind the post-reperfusion inflammatory burst remain unclear, hindering predictable therapeutic outcomes. This study reveals that the serine de novo synthesis pathway, with PHGDH as the key enzyme, is significantly downregulated after cerebral ischemia-reperfusion. PHGDH, highly and specifically expressed in brain astrocytes and downregulated post-I/R, when inhibited in astrocytes, exacerbates I/R-induced pathological damage, impairs sensory and motor functions, and promotes AIM2 inflammasome activation and pyroptosis. Mechanistically, PHGDH maintains electron transport chain integrity and mitochondrial homeostasis by facilitating heme and ubiquinone synthesis, reducing ROS generation and inhibiting AIM2 inflammasome activation and pyroptosis, thereby decreasing inflammatory cytokine release and protecting neurons from I/R injury. Conversely, PHGDH inhibition disrupts astrocytic redox balance and electron transport chain integrity, amplifying oxidative stress, releasing abundant dsDNA into the cytoplasm, inducing AIM2 inflammasome activation and pyroptosis, and promoting neuronal injury (Fig. 9). This study identifies the PHGDH-mediated serine de novo synthesis pathway as a critical metabolic hub regulating astrocytic pyroptosis after cerebral I/R, linking metabolism, oxidative stress, and pyroptosis to expand the understanding of pyroptosis regulatory mechanisms and offering potential therapeutic targets for ischemic stroke by targeting this metabolic-inflammatory axis.

Neuroinflammation is a pivotal process in cerebral ischemia-reperfusion injury, with inflammasome activation playing a central role. First introduced by Martinon et al., in 2002 [29,30], inflammasomes are multi-protein complexes where NLR family proteins (e.g., NLRP1, NLRP3), AIM2, or pyrin sense pathogen/damage-associated molecular patterns (PAMPs/DAMPs), recruiting the adaptor protein ASC to assemble complexes that activate caspase-1. Activated caspase-1 cleaves GSDMD into its pore-forming N-terminal fragment, inducing pyroptotic cell lysis and release of pro-inflammatory cytokines like IL-1β and IL-18. In ischemic stroke, multiple inflammasomes (NLRC4, NLRP1, NLRP3, NLRP6, AIM2) have been implicated [31], with AIM2 uniquely recognizing cytosolic dsDNA to drive AIM2-ASC-caspase-1 complex formation [27] and GSDMD-mediated pyroptosis [28]. While recent studies have linked dsDNA-driven cGAS-STING signaling to AIM2 inflammasome activation in retinal ganglion cell death [32] and identified Lcn2-24p3R pathways in astrocytic pyroptosis after I/R^8^, the upstream regulators of AIM2 in astrocytes during cerebral ischemia-reperfusion remain poorly understood. Here, we show that cerebral I/R significantly upregulates AIM2 and GSDMD expression, accompanied by coordinated activation of AIM2 inflammasome components and robust release of IL-1β/IL-18. Mechanistically, mitochondrial damage following I/R enhances ROS production and dsDNA release, triggering AIM2 inflammasome activation in astrocytes. This cascade promotes astrocytic pyroptosis, neuronal injury, and exacerbation of sensorimotor deficits in mice, highlighting AIM2 as a critical mediator of post-ischemic neuroinflammation with uncharacterized upstream metabolic and mitochondrial dependencies.

In our mechanistic studies, we further elucidated the relationship between PHGDH and pyroptosis in astrocytes following ischemia-reperfusion. Through RNA sequencing, we identified significant downregulation of serine, glycine, and threonine metabolic pathways in cerebral tissue after I/R. As previously documented, serine and glycine are among the least abundant amino acids in the brain microenvironment, with concentrations 4 to 50 times lower than those in plasma [33]. Glycine biosynthesis predominantly relies on SHMT1 and SHMT2 for production from serine, whereas PHGDH, serving as the rate-limiting enzyme in de novo serine synthesis, plays an indispensable role in maintaining amino acid homeostasis, one-carbon metabolism, and the biosynthesis of pyrimidines and purines in brain cells. Our RNA sequencing data further revealed that PHGDH knockdown significantly diminished SHMT1 and SHMT2 expression levels, underscoring the non-redundant role of PHGDH in cerebral serine and glycine synthesis.

Serine metabolism has been extensively investigated in neurodegenerative diseases. Genetic deficiencies in serine biosynthesis pathway enzymes are associated with human Neu-Laxova syndrome [34], a rare disorder characterized by severe peripheral and central nervous system malformations leading to prenatal or early postnatal mortality, for which exogenous serine supplementation represents an established therapeutic strategy. Juliette et al. demonstrated that early impairment of l-serine synthesis in astrocytes of Alzheimer's disease models induces cognitive deficits, synaptic plasticity impairment, and spatial memory dysfunction [17]. In advanced disease stages, reduced PHGDH expression coincides with proposed dietary serine supplementation as a potential therapeutic approach. In the brain, astrocytes synthesize l-serine in a PHGDH-dependent manner and transfer it to neurons to sustain d-serine production, which modulates NMDAR activity. Inhibition of PHGDH activity or knockdown of its expression attenuates NMDAR-mediated synaptic potentials and long-term potentiation (LTP) in hippocampal neurons [35]. These findings align with our results showing that PHGDH knockdown in astrocytes or exogenous deprivation of serine and glycine exacerbates cerebral I/R injury and impairs neurological function, highlighting the critical role of serine-glycine metabolism in neuroprotection against ischemic insult.

PHGDH-mediated serine de novo synthesis pathway exhibits a close association with the immune system. Preclinical studies have demonstrated that PHGDH knockdown, enzymatic activity inhibition, or serine deprivation enhances IFNβ-mediated antiviral innate immunity [23]. In macrophages, PHGDH loss or serine deficiency regulates the IGF1-p38 signaling axis to induce M1 macrophage polarization [21], thereby promoting the release of pro-inflammatory mediators such as IL-1β. In tumor-associated macrophages (TAMs), PHGDH expression is significantly upregulated; inhibition of PHGDH disrupts cellular bioenergetics and mitochondrial respiration, suppresses mTORC1 pathway activation, and attenuates the M2 phenotype transition [36]. These findings align with our results, demonstrating that PHGDH knockdown enhances innate immune responses and pro-inflammatory cytokine expression. RNA sequencing further revealed that PHGDH inhibition in astrocytes significantly upregulated the pattern recognition receptor signaling pathway, promoting the expression of AIM2, GSDMD, and the secretion of IL-1β/IL-18.

Our mechanistic experiments showed that combined serine and glycine deprivation exerts a synergistic effect in promoting astrocyte pyroptosis following I/R. Notably, individual depletion of serine or glycine alone did not exacerbate pyroptosis in astrocytes, potentially due to the metabolic interconversion between these two amino acids. Importantly, serine supplementation following PHGDH knockdown partially rescued I/R-induced astrocyte pyroptosis, suggesting that PHGDH may mediate additional functions beyond its canonical role in serine biosynthesis. Our metabolomics analysis and functional experiments demonstrated that PHGDH knockdown in astrocytes following I/R significantly reduced intracellular GSH levels, elevated the GSSG/(GSH + GSSG) redox ratio, and decreased the NADP+/NADPH ratio compared to the shCtrl group. Concomitantly, PHGDH-depleted astrocytes exhibited a marked increase in ROS production, indicative of severe oxidative stress. Exogenous supplementation with the antioxidant NAC significantly attenuated ROS generation and rescued astrocyte viability, linking PHGDH loss to redox imbalance. Given PHGDH's central role in cellular metabolism, we further investigated the metabolic repercussions of its depletion. Metabolomics profiling revealed profound reductions in heme and ubiquinone levels, with heme nearly undetectable in PHGDH-deficient cells. These molecules are critical components of the ETC in oxidative phosphorylation. Notably, PHGDH has been reported to interact with UQCRFS1 [37], an essential subunit of ETC complex III. Transcriptomic analysis corroborated these findings by showing significant downregulation of most genes involved in heme biosynthesis. Functional assays confirmed that PHGDH knockdown significantly impaired ETC complex III activity and increased dsDNA release in astrocytes. These results align with prior studies demonstrating that PHGDH knockdown in endothelial cells induces mitochondrial autophagy, depletes heme, and promotes cell death [38]. Collectively, these data indicate that PHGDH loss disrupts mitochondrial homeostasis by compromising the oxidative respiratory chain, thereby exacerbating ROS production and dsDNA-mediated activation of the AIM2 inflammasome in a synergistic manner.

We envision that drugs targeting PHGDH could be developed in future studies for the treatment of cerebral ischemia-reperfusion injury. If successfully achieved, their safety and efficacy cannot be overlooked. In our current study, we have confirmed that astrocyte-specific PHGDH overexpression can significantly improve neurological function and motor balance ability in mice after I/R, as well as inhibit the progression of pyroptosis and inflammation following I/R. Notably, astrocyte-specific PHGDH overexpression did not impair neurological function scores, motor function, or balance ability in Sham-operated mice, nor did it promote inflammation. Therefore, targeting PHGDH is effective and safe in animal models, and it can serve as a potential therapeutic target for cerebral ischemia-reperfusion injury. However, due to species differences between mice and humans, the mouse model may not fully simulate the state of cerebral ischemia-reperfusion in the human brain, which necessitates further series of studies in the future. Nevertheless, a multi-omics study on the overexpression of de novo serine synthesis pathway enzymes in pluripotent stem cell (iPSC)-derived human astrocytes under physiological conditions can provide a reference in the context of human models. In that study, PHGDH overexpression increases the production of reducing substances such as glutathione, and enhances mitochondrial oxidative phosphorylation and ATP synthesis [39], which is consistent with the findings of our study. In summary, targeted PHGDH overexpression and activation hold promise as a potential key target for the treatment of cerebral ischemia-reperfusion injury in the future.

Existing studies on PHGDH overexpression or activation as a therapeutic approach remain scarce. Clioquinol (CQ), a PHGDH agonist that targets and activates PHGDH. Research showed that when PHGDH agonists are administered to patients with refractory epilepsy, both the duration of epileptic seizures and the severity of seizures are reduced, with no adverse reactions such as cognitive impairment reported [40]. The combined use of small molecule kinase inhibitors (SMKIs) can upregulate the expression of PHGDH. In dilated cardiomyopathy, the application of SMKIs can restore the serine-glycine-one-carbon metabolic cycle, improve mitochondrial respiratory function and ATP production, and alleviate myocardial cell contractile dysfunction and metabolic disorders in dilated cardiomyopathy [41].

In summary, this study integrates transcriptomic, metabolomic, and functional experimental data to elucidate the role of PHGDH-mediated serine de novo synthesis in regulating astrocyte oxidative stress and pyroptosis following cerebral ischemia-reperfusion. Our findings not only confirm that cerebral I/R induces astrocyte pyroptosis via AIM2 inflammasome activation but also establish a mechanistic link whereby PHGDH deficiency disrupts mitochondrial homeostasis, leading to enhanced release of ROS and dsDNA. These mitochondrial damage-associated molecular patterns synergistically activate the AIM2-ASC-Caspase-1 signaling axis, promoting astrocyte pyroptotic cell death and the subsequent release of pro-inflammatory cytokines IL-1β and IL-18. This cascade exacerbates neuroinflammatory responses and accelerates the progression of cerebral I/R injury. Collectively, these results highlight the metabolism-inflammation axis as a potential therapeutic target, suggesting that combined modulation of pyroptosis via inflammasome inhibitors and serine metabolism via PHGDH agonists may represent a novel strategy to mitigate neuroinflammation after cerebral ischemia-reperfusion. Additionally, alterations in serine metabolites (e.g., serine, glycine) in cerebrospinal fluid and plasma could serve as valuable clinical biomarkers for monitoring neuroinflammatory severity in I/R patients.

## Ethics approval and consent to participate

All animal experiments were carried out in accordance with protocols approved by the Institutional Ethics Committee of the Xijing Hospital. All experimental procedures were approved by the Institutional Animal Care and Use Committee of Air Force Military Medical University.

## Consent for publication

Not applicable.

## Funding

This work was supported by the National Natural Science Foundation of China (No.81971227), Key Research and Development Program of Shaanxi Province (2018ZDXM-SF-086 and 2024SF-YBXM-166), Key Laboratory Project of Shaanxi Province (2025SYS-SYSZD-028), National Natural Science Foundation of China (82401531), the China Postdoctoral Science Foundation, No.18 Special Fund (2025T181193).

## CRediT authorship contribution statement

**Jimeng Zhang:** Conceptualization, Data curation, Formal analysis, Investigation, Methodology, Project administration, Supervision, Validation, Writing – original draft. **Yaowen Luo:** Data curation, Investigation, Methodology. **Changcai Xie:** Data curation, Investigation. **Min Zhang:** Investigation. **Heyao Qin:** Investigation. **Yuefei Zhou:** Investigation. **Xiuquan Wu:** Investigation. **Chenchen Hu:** Investigation. **Feiming Hu:** Investigation. **Xiaowei Fei:** Funding acquisition, Investigation. **Hongchen Zhang:** Funding acquisition, Investigation. **Juan Li:** Investigation. **Yihao Fu:** Investigation. **Yunchao Yuan:** Investigation. **Shuya Yang:** Conceptualization, Data curation, Formal analysis, Funding acquisition, Investigation, Methodology, Project administration, Supervision. **Dakuan Gao:** Conceptualization, Data curation, Formal analysis, Funding acquisition, Investigation, Methodology, Project administration, Resources, Software, Supervision, Validation, Visualization, Writing – original draft, Writing – review & editing.

## Declaration of competing interest

The authors declare that they have no known competing financial interests or personal relationships that could have appeared to influence the work reported in this paper.

## Data Availability

No data was used for the research described in the article.

## Abbreviations

PHGDH: Phosphoglycerate dehydrogenase
MCAO/R: Middle cerebral artery occlusion/reperfusion
OGD/R: Oxygen-glucose deprivation/reoxygenation
AAV: Adeno-associated virus
CCA: Common carotid artery
ECA: External carotid artery
ICA: Internal carotid artery
MCA: Middle cerebral artery
mNSS: Modified neurological severity score
DMEM: Dulbecco's Modified Eagle Medium
WB: Western blotting
IF: Immunofluorescence
TUNEL: Terminal deoxynucleotidyl transferase dUTP nick end labeling
DAMPs: Damage-associated molecular patterns
GSDMD: Gasdermin D
SSP: Steady-state PCR
TCA: Tricarboxylic acid cycle
I/R: Ischemia/reperfusion
KD: Knockdown
ELISA: Enzyme-linked immunosorbent assay
ROS: Reactive oxygen species
DEGs: Differentially expressed genes
TEM: Transmission electron microscopy
ANOVA: Analysis of variance
TLR: Toll-like receptor
NLR: Nod-like receptor
SHMT: Serine hydroxymethyltransferase
KEGG: Kyoto Encyclopedia of Genes and Genomes
GO: Gene Ontology
HE: Hematoxylin and eosin staining
LV: Lentivirus
GSH: Glutathione
NAC: N-Acetylcysteine
DMSO: Dimethyl sulfoxide
NADPH: Nicotinamide adenine dinucleotide phosphate
dsDNA: Double-stranded DNA
