# Supplementary material for: PHGDH-dependent serine metabolism in astrocytes: A key regulator of oxidative stress and pyroptosis in cerebral ischemia-reperfusion injury
Source: Redox Biol. 2025 Nov 29;89:103954. doi: 10.1016/j.redox.2025.103954 (PMC12721196; doi:10.1016/j.redox.2025.103954)
Supplement: Multimedia component 1 [file mmc1.docx]

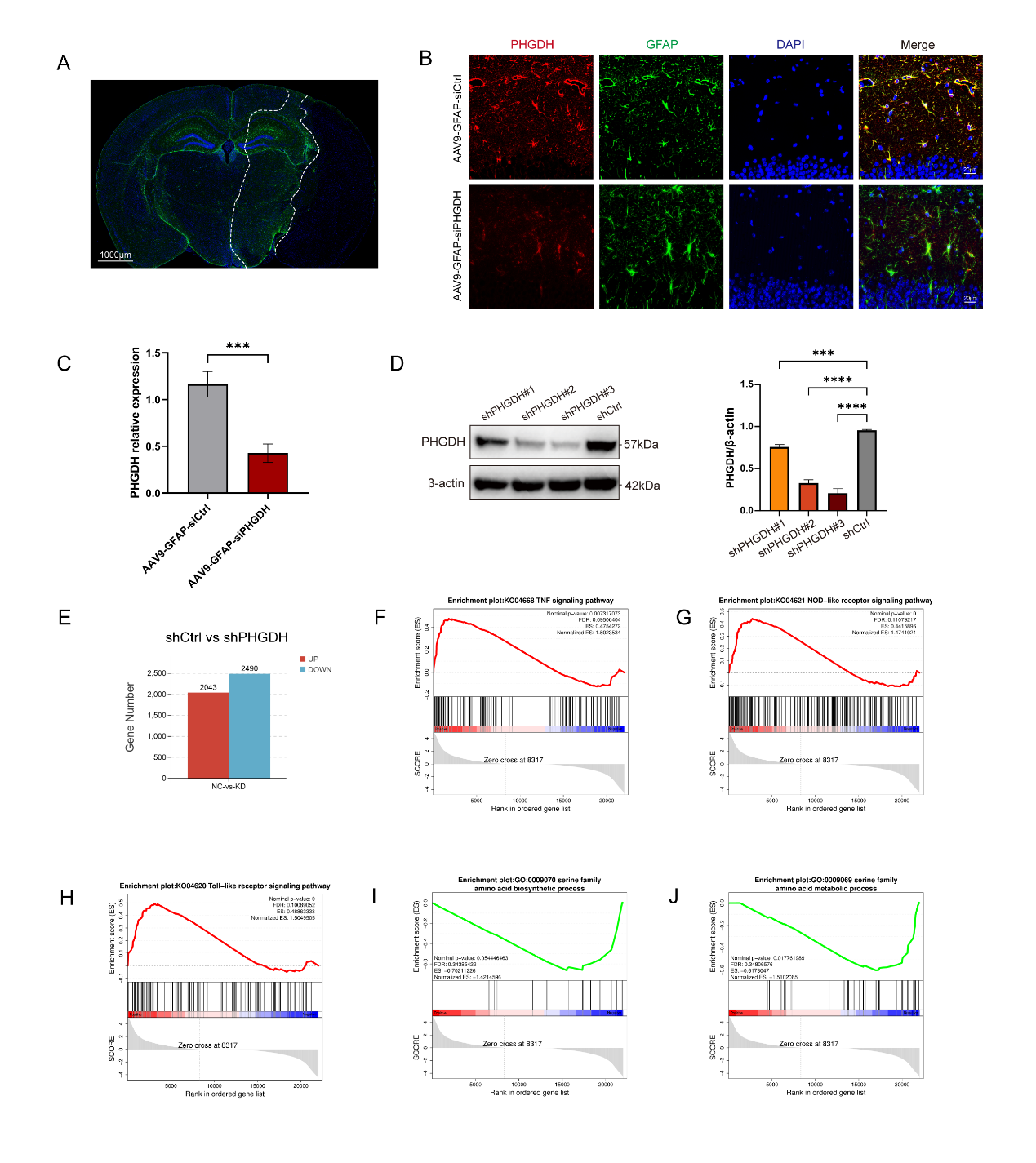


**Fig. S1** (A) Astrocytes are activated following middle cerebral artery occlusion/reperfusion (MCAO/R). Scale bars = 1000 μm. (B) Immunofluorescence staining of the hippocampal region at 4 weeks after injection of AAV9-GFAP-siCtrl or AAV9-GFAP-siPHGDH. Scale bars = 20 μm. (C) Quantitative analysis of immunofluorescence staining. (D) Western blot validation of astrocytes transfected with lentivirus. (E) Statistical analysis of transcriptomic gene modulation. (F-J) Gene Set Enrichment Analysis (GSEA) of transcriptomic data. Statistical analysis was performed using one-way ANOVA or *t*-test. Each experiment was repeated independently at least three times. Values are expressed as mean ± SD. **P < 0.05* indicates a statistically significant difference between the two groups. **P<0.05, **P<0.01, ***P<0.001, ****P<0.0001.*


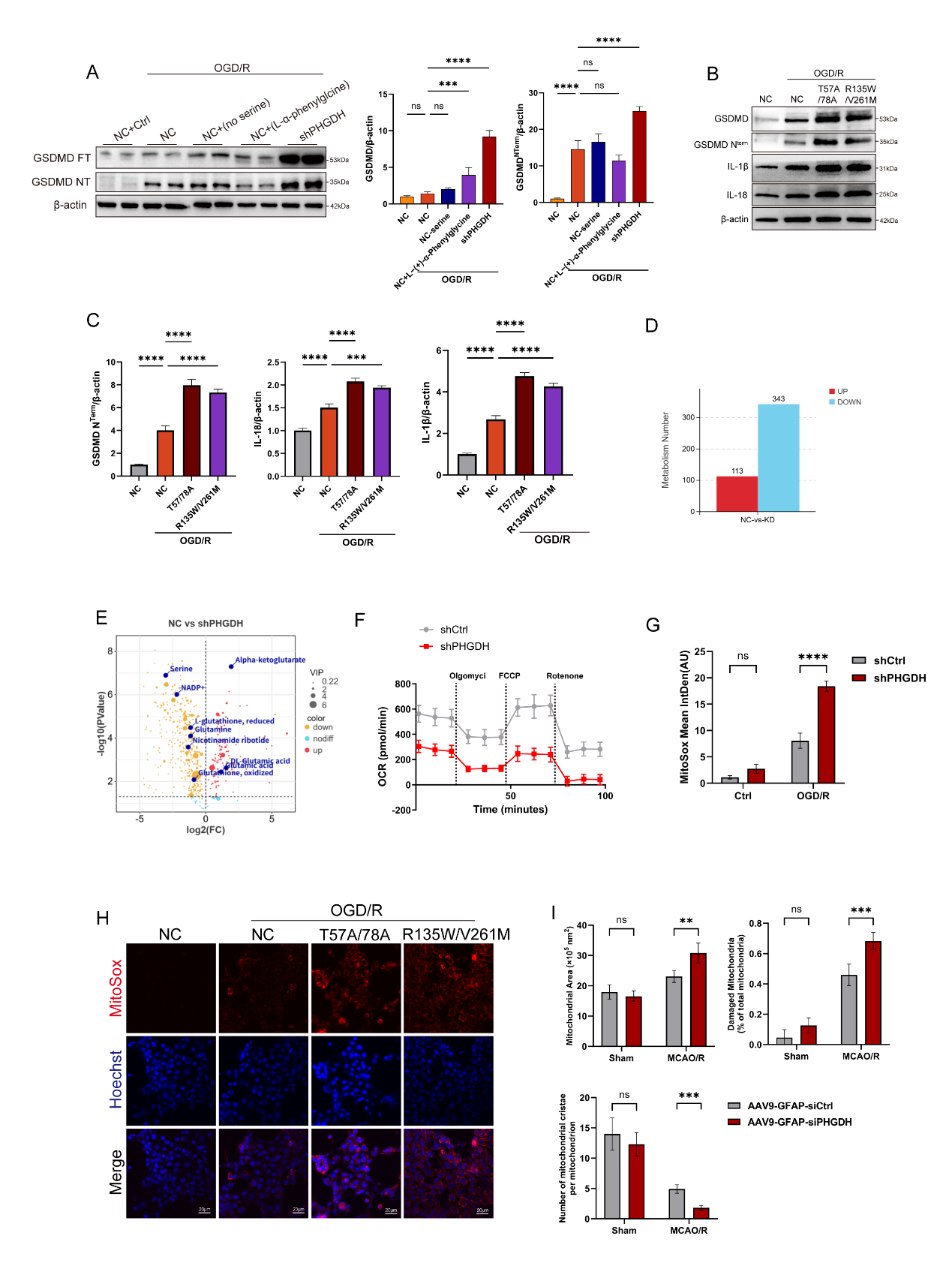


**Fig. S2** (A) Western blot analysis of the effects of conditional serine deficiency and L-α-phenylglycine treatment on GSDMD expression in astrocytes after oxygen-glucose deprivation/reoxygenation (OGD/R). (B, C) Effects of PHGDH mutants (T57A/78A and R135W/V261M) on the expression of GSDMD, GSDMD^Nterm^, IL-1β, and IL-18 in astrocytes after OGD/R. (D) Statistical analysis of transcriptomic gene changes. (E) Volcano plot of metabolites related to oxidative stress from transcriptomic sequencing data. (F) Statistical analysis of oxygen consumption rate (OCR) over time in mitochondrial stress tests. (G) Quantitative analysis of MitoSox staining. (H) Effects of PHGDH mutants (T57A/78A and R135W/V261M) on MitoSox levels in astrocytes after OGD/R. Scale bars = 20μm. (I) Statistics of mitochondrial area, mitochondrial damage ratio, and mitochondrial cristae number ratio in Figure 7L. Statistical analysis was performed using one-way ANOVA, two-way ANOVA or *t*-test. Each experiment was repeated independently at least three times. Values are expressed as mean ± SD. **P < 0.05* indicates a statistically significant difference between the two groups. **P<0.05, **P<0.01, ***P<0.001, ****P<0.0001.*


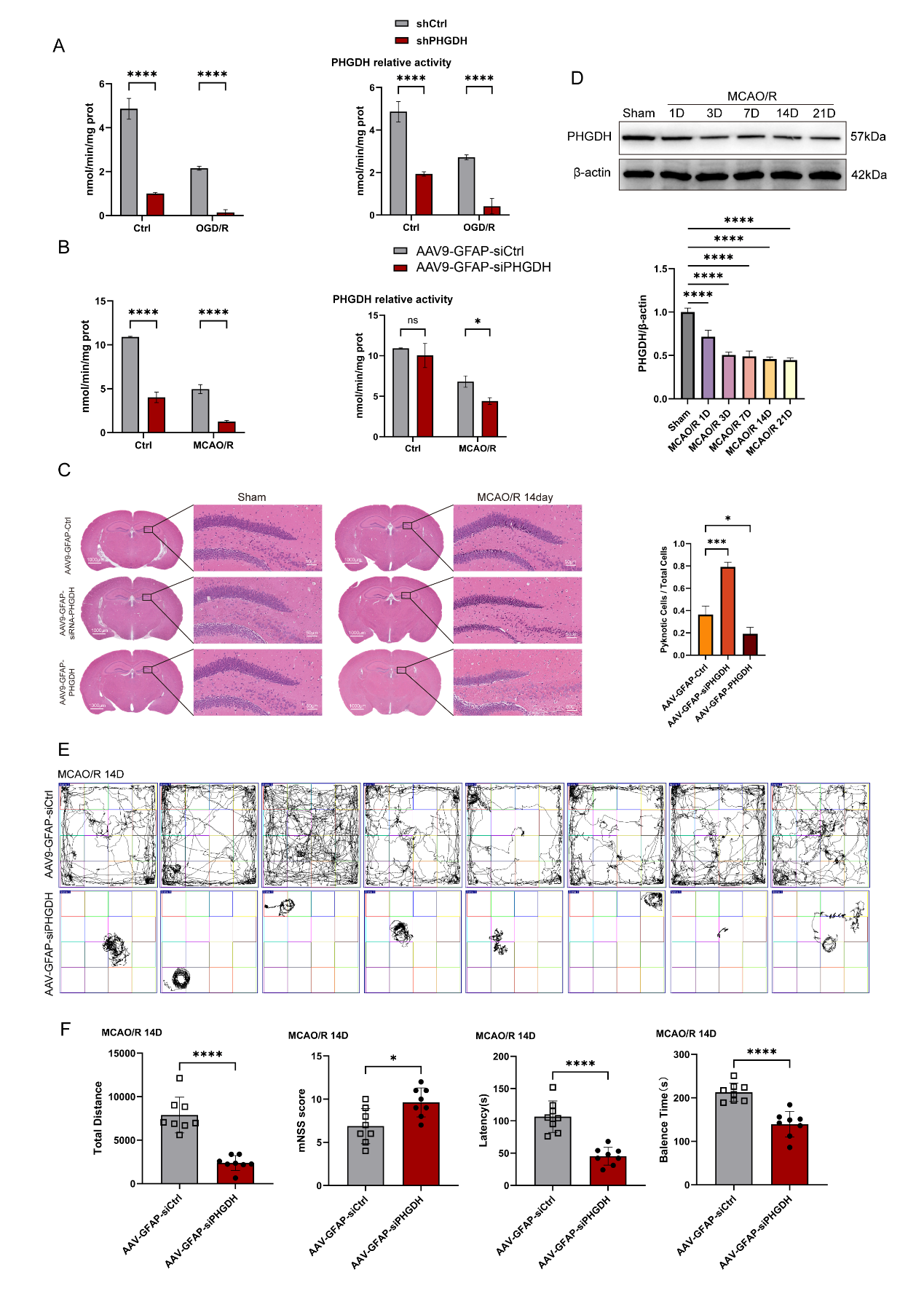


**Fig. S3** (A) PHGDH activity assay in shCtrl and shPHGDH astrocytes with or without OGD/R treatment. (B) PHGDH activity assay in astrocyte-specific PHGDH-knockdown and control groups with or without MCAO/R treatment. (C) HE staining of AAV9-GFAP-Ctrl, AAV9-GFAP-siPHGDH, and AAV9-GFAP-PHGDH groups at 14 days after MCAO/R. Scale bars = 1000 μm or 50 μm. (D) Western blot analysis of PHGDH protein levels at 1, 3, 7, 14, and 21 days after MCAO/R. (E) Open field behavioral test of mice in AAV9-GFAP-siCtrl and AAV9-GFAP-siPHGDH groups at 14 days after MCAO/R. (F) Statistical analysis of total movement distance in the open field test, mNSS scores, rotarod test latency, and balance time. Statistical analysis was performed using one-way ANOVA, two-way ANOVA or *t*-test. Each experiment was repeated independently at least three times. Values are expressed as mean ± SD. **P < 0.05* indicates a statistically significant difference between the two groups. **P<0.05, **P<0.01, ***P<0.001, ****P<0.0001.*


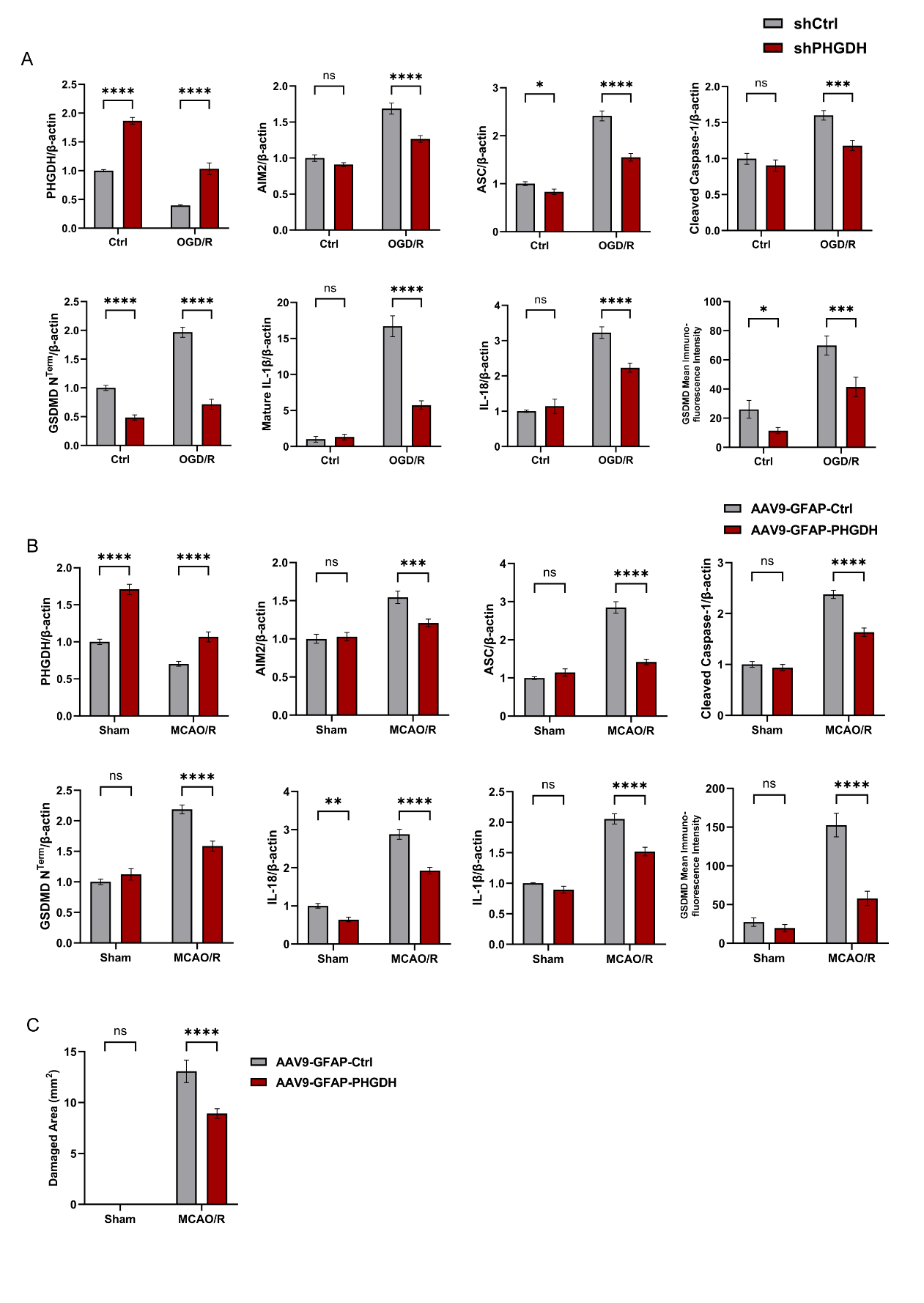


**Fig. S4** (A) Western blot analysis of PHGDH, AIM2, ASC, Cleaved Caspase-1, GSDMD^Nterm^, IL-1β, and IL-18 protein levels in figure 8A, and quantitative analysis of fluorescence intensity for GSDMD immunofluorescence staining in figure 8B. (B) Western blot analysis of PHGDH, AIM2, ASC, Cleaved Caspase-1, GSDMD^Nterm^, IL-1β, and IL-18 protein levels in figure 8D, and quantitative analysis of fluorescence intensity for GSDMD immunofluorescence staining in figure 8E. (C) Quantitative analysis of lesion area in HE staining in figure 8F. Statistical analysis was performed using two-way ANOVA. Each experiment was repeated independently at least three times. Values are expressed as mean ± SD. **P < 0.05* indicates a statistically significant difference between the two groups. **P<0.05, **P<0.01, ***P<0.001, ****P<0.0001.*


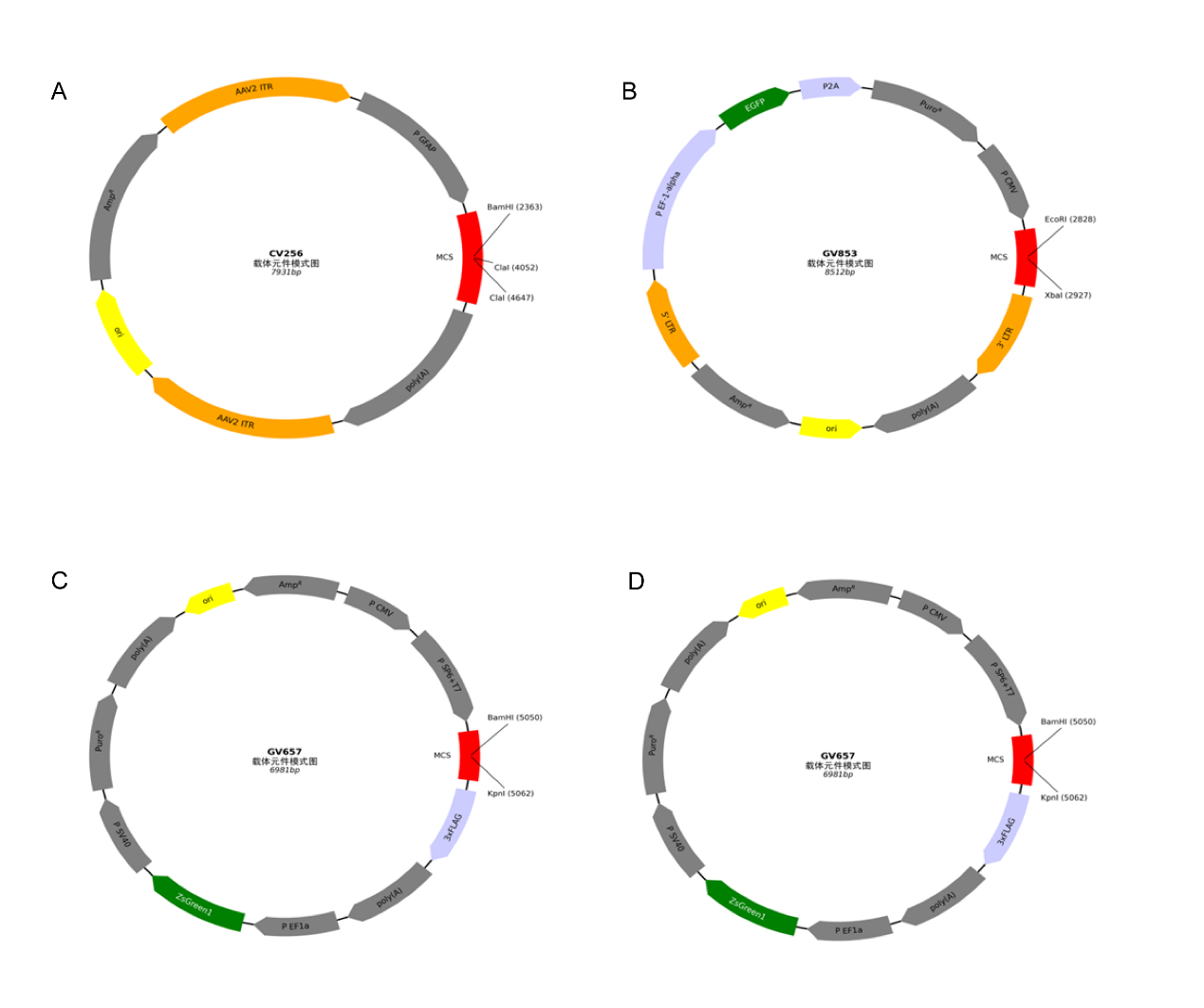


**Fig. S5** (A) Schematic diagram of AAV9-GFAP-PHGDH vector construction. (B) Schematic diagram of LV-PHGDH vector construction. (C) Schematic diagram of PHGDH mutant (R135W/V261M) vector construction. (D) Schematic diagram of PHGDH mutant (T57A/78A) vector construction.
